# Endocrine and metabolic impacts of warming aquatic habitats: differential responses between recently isolated populations of a eurythermal desert pupfish

**DOI:** 10.1093/conphys/cow047

**Published:** 2016-11-03

**Authors:** Sean C Lema, Michelle I Chow, Emily J Resner, Alex A Westman, Darran May, Andrew H Dittman, Kristin M Hardy

**Affiliations:** 1 Biological Sciences Department, Center for Coastal Marine Sciences, California Polytechnic State University, San Luis Obispo, CA 93407, USA; 2 School of Aquatic and Fishery Sciences, University of Washington, Seattle, WA 98105, USA; 3 Environmental and Fisheries Sciences Division, Northwest Fisheries Science Center, National Marine Fisheries Service, NOAA, Seattle, WA 98112, USA

**Keywords:** Climate change, fish, lactate dehydrogenase, metabolism, temperature, thyroid hormone

## Abstract

Temperatures of inland aquatic habitats are increasing with climate change, and understanding how fishes respond physiologically to thermal stress will be crucial for identifying species most susceptible to these changes. Desert fishes may be particularly vulnerable to rising temperatures because many species occupy only a fraction of their historical range and occur in habitats with already high temperatures. Here, we examined endocrine and metabolic responses to elevated temperature in Amargosa pupfish, *Cyprinodon nevadensis amargosae*. We studied *C. n. amargosae* from two habitats with distinct thermal conditions: the Amargosa River, which experiences diurnally and seasonally variable temperatures (0.2–40°C); and Tecopa Bore, a spring and marsh fed by hot groundwater (47.5°C) from an artesian borehole. These allopatric populations differ in morphology, and prior evidence suggests that temperature might contribute to these differences via altered thyroid hormone (TH) regulation of morphological development. Here, we document variation in hepatic iodothyronine deiodinase type 2 (*dio2*) and type 3 (*dio3*) and TH receptor β (*trβ*) gene transcript abundance between the Amargosa River and Tecopa Bore wild populations. Fish from these populations acclimated to 24 or 34°C retained differences in hepatic *dio2*, *dio3* and *trβ* mRNAs and also varied in transcripts encoding the TH membrane transporters monocarboxylate transporter 8 (*mct8*) and organic anion-transporting protein 1c1 (*oatp1c1*). Tecopa Bore pupfish also exhibited higher *dio2* and *trβ* mRNA levels in skeletal muscle relative to Amargosa River fish. Muscle citrate synthase activity was lower at 34°C for both populations, whereas lactate dehydrogenase activity and lactate dehydrogenase A-chain (*ldhA*) transcripts were both higher and 3,5,3′-triiodothryonine responsive in Tecopa Bore pupfish only. These findings reveal that local population variation and thermal experience interact to shape how pupfish respond to elevated temperatures, and point to the need to consider such interactions in management actions for desert fishes under a changing climate.

## Teaser

Imperilled desert fishes occupying isolated habitats that limit dispersal are vulnerable to temperature alterations because of a changing climate. Here, we identify differences in temperature effects on thyroid hormone signalling, anaerobic metabolism and thyroid hormone-mediated metabolic gene expression between recently isolated populations of a desert pupfish.

## Introduction

Inland aquatic habitats are warming more rapidly than either the oceans or the atmosphere under a changing global climate (Schneider *et al*., 2009; Layden *et al*., 2015; O'Reilly *et al*., 2015). Such rising temperatures are expected to alter freshwater fish habitat use, shift species’ ranges and life histories, and induce a suite of physiological responses to high temperature (Wood and McDonald, 1997; Roessig *et al*., 2004; Ficke *et al*., 2007). Understanding how fish respond physiologically to elevated temperature—and how these responses vary among taxa or populations—will be crucial for predicting how fish communities will change in the face of warming habitats (Pörtner and Farrell, 2008), as well as for evaluating whether species persistence will be aided by plasticity in thermal tolerance (Hofmann and Todgham, 2010; Bozinovic and Pörtner, 2015).

Pupfishes (genus *Cyprinodon*) of the North American southwestern deserts provide a model set of taxa for examining the fundamental mechanisms for coping physiologically with high temperatures. Pupfish in this region evolved some of the highest critical thermal maxima and broadest thermal tolerances recorded in freshwater fishes (Beitinger *et al*., 2000; Motani and Wainwright, 2015), with several species of pupfishes surviving temperatures exceeding 42°C (Brown and Feldmeth, 1971; Otto and Gerking, 1973; Feldmeth, 1981). In this study, we examined the influences of temperature on the endocrine physiology and metabolism of Amargosa pupfish, *Cyprinodon nevadensis amargosae*, from the Death Valley region of California and Nevada, USA. This extreme desert region is home to a clade of three pupfish species partitioned into eight subspecies (Miller, 1948; Soltz and Naiman, 1978), which appear to have evolved within the last 5000–20 000 years (Martin *et al*., 2016). Similar to most other pupfishes through the North American desert southwest, Death Valley's pupfishes occupy restricted geographical ranges and are isolated in remote groundwater-fed springs, desert streams or marshes (Soltz and Naiman, 1978). Isolation in such small, discrete habitats has made pupfishes in this region vulnerable to human activities (Fagan *et al*., 2002). One pupfish from the Death Valley region is already extinct (Miller *et al*., 1989), and several other pupfishes from this clade have ‘threatened’ or ‘endangered’ legal status (Moyle *et al*., 2011).

Amargosa pupfish occur in several habitats within the Death Valley region, including ~13 km of permanent fluvial habitat in the Amargosa River and a small artesian spring referred to as Tecopa Bore, where water emerges from the ground at 47.5°C and cools gradually as it flows through an associated marsh (Naiman, 1974). This Tecopa Bore habitat was created inadvertently in 1967 as a result of commercial mineral drilling, and *C. n. amargosae* pupfish from the Amargosa River dispersed into the habitat shortly thereafter. Pupfish are found in areas of the Tecopa Bore habitat at temperatures exceeding 36°C, although preferred temperatures may be closer to 30°C (McCauley and Thomson, 1988). In contrast, the Amargosa River is a desert stream with temperature conditions that vary from near freezing to >40°C seasonally and can change more than 25°C diurnally (Soltz and Naiman, 1978). Recent examination of these two populations of *C. n. amargosae* found that pupfish in the extreme thermal environment of Tecopa Bore are not only smaller in length and mass than conspecifics from the Amargosa River, but that Tecopa Bore fish also differ in body shape and frequently lack pelvic fins (Caldwell *et al*., 2015). A previous study found that pharmacological inhibition of endogenous thyroid hormone (TH) production during larval life induces *C. n. amargosae* to develop a proportionally larger head and eye, a reduced body depth, and no paired pelvic fins (Lema and Nevitt, 2006). *Cyprinodon nevadensis amargosae* in Tecopa Bore mirror this same morphology, with a proportionally large head and eye, reduced body depth, and lower occurrence of pelvic fins (Caldwell *et al*., 2015). Notably, larval pupfish exposed to elevated temperatures are also less likely to develop paired pelvic fins (Lema and Nevitt, 2006; Lema, 2008, 2014). In teleost fishes, pelvic fins develop during the transition from a larval life stage to a juvenile fish (Yamanoue *et al*., 2010), and this developmental change is mediated in part by THs (Brown, 1997). Given that evidence, it is possible that the observed morphological differences between the Amargosa River and Tecopa Bore populations might arise in part from altered TH physiology in Tecopa Bore pupfish because of the consistently high temperatures of their habitat.

Thyroid hormone signalling might also play an important role in metabolic acclimation to temperature variation in teleost fishes (Little *et al*., 2013), as it does in mammals (Silva, 1995; Yen, 2001; Mullur *et al*., 2014). Zebrafish (*Danio rerio*) acclimated to 18 or 28°C and treated with a pharmacological goitrogen to inhibit endogenous TH production subsequently exhibited dissimilar swimming performance, metabolic scope and metabolism-associated gene expression in patterns indicative of impaired TH-mediated metabolic regulation at warm temperature (Little *et al*., 2013). Several studies in fishes have also found that exposure to elevated temperatures can alter TH status (Leatherland *et al*., 1977, 1980; Lee *et al*., 2014). For instance, rainbow trout (*Oncorhynchus mykiss*) acclimated to 11°C and transferred to 19°C exhibited reduced plasma thyroxine (T_4_; the primary circulating TH in teleost fishes) compared with trout transferred to 5°C (Eales *et al*., 1982). This reduction in T_4_ at 19°C was accompanied by a 4-fold increase in T_4_ metabolic clearance and conversion to 3,5,3′-triiodothryonine (T_3_), a more active structural form of TH (Eales *et al*., 1982). Thyroid hormone conversion occurs via the action of iodothyronine deiodinase enzymes, with type II deiodinase (Dio2) mediating outer-ring deiodination (ORD) activity to convert T_4_ to T_3_, and type III deiodinase (Dio3) catalysing inner-ring deiodination (IRD) to convert T_4_ to the less active THs reverse T_3_ (rT_3_) or 3,5-diiodothyronine (T_2_; Köhrl, 2000; Orozco and Valverde, 2005). In fish, there is evidence that deiodinase activity may vary in patterns linked to temperature. For instance, in trout liver microsomes, rates of ORD of T_4_ were 2.4-fold higher at 12–13°C and 2.8-fold higher at 18°C than at 4°C (Johnston and Eales, 1995).

In this study, we examined whether temperature experience alters TH physiology and metabolism in patterns that help to explain the observed phenotypic differences between the Amargosa River and Tecopa Bore populations of *C. n. amargosae*. First, we tested whether these two populations differ in TH physiology by quantifying plasma T_4_ and hepatic gene transcript abundance for deiodinase enzymes type I (*dio1*), type II (*dio2*) and type III (*dio3*), as well as TH receptors (*trαA*, *trαB* and *trβ*) in the wild. Based on the morphological variation between the populations and our previous experimental findings of how food availability and temperature alter morphological development in pupfish (e.g. Lema and Nevitt, 2006; Caldwell *et al*., 2015), we hypothesized that *C. n. amargosae* in Tecopa Bore would exhibit characteristics resembling a hypothyroid state, including reduced plasma T_4_ and higher hepatic *dio2* mRNA levels. Second, we hypothesized that any observed population differences in TH physiology between the Amargosa River and Tecopa Bore populations would be caused predominantly by the differing temperature experience of these populations. We tested this idea by acclimating pupfish from both populations to 24 or 34°C in captivity for 88 days. At the end of this 88 day thermal acclimation period, we administered exogenous T_3_ for 18–24 h to a subset of pupfish from both populations and temperature treatments to activate T_3_-regulated changes in gene expression and metabolism. Liver and skeletal muscle tissues were examined for differences in the relative abundance of gene transcripts encoding TH signalling-associated proteins, including deiodinase enzymes, TH receptors and the TH membrane transporters monocarboxylate transferase 8 (*mct8*) and organic anion-transporting polypeptide 1c1 (*oatp1c1*; Muzzio *et al*., 2014). Citrate synthase (CS) activity was also quantified as an indicator of aerobic metabolic capacity, and lactate dehydrogenase (LDH) activity was examined as an indicator of anaerobic metabolic capacity. The relative abundance of mRNAs encoding several metabolism-linked proteins was also quantified to evaluate T_3_ induction of metabolic gene expression.

## Materials and methods

### Study populations

*Cyprinodon nevadensis amarogsae* pupfish were studied from two allopatric populations, the Amargosa River (35°51.275′N, 116°13.833′W) and Tecopa Bore (35°53.140′N, 116°14.050′W), located in the Death Valley desert region of eastern California, USA. All procedures were approved by the Animal Care and Use Committee of California Polytechnic State University (protocol #1507).

### Animal collection

On 9 May 2014, adult *C. n. amargosae* pupfish were collected from the Amargosa River and Tecopa Bore habitats using minnow traps. A subset of pupfish from the Amargosa River population (*n =* 9 fish of each sex) and from Tecopa Bore (*n =* 8 males and *n =* 11 females) were immediately euthanized (tricaine methansulfonate, MS222; Argent Chemicals, Inc.), and then length and mass were measured. Blood was collected and centrifuged, and plasma stored for subsequent T_4_ quantification. Liver tissues were dissected and immersed in RNAlater^®^ (ThermoScientific) at 4°C for 48 h before being stored at −20°C.

Additional adult *C. n. amargosae* pupfish collected from the Amargosa River and Tecopa Bore on 9 May 2014 were transported to California Polytechnic State University, San Luis Obispo, CA, USA. After collection, fish were maintained in 208 litre holding tanks to allow for acclimation to captive conditions. Pupfish from the Amargosa River were maintained at 24°C, and Tecopa Bore pupfish at 34°C. For the duration of the study, all fish were fed a 1:1 mixture of commercial spirulina (Aquatic Eco-Systems, Inc., Apopka, FL, USA) and brine shrimp (San Francisco Bay Brand, Inc., Newark, CA, USA) flake feeds *ad libitum* twice daily and were maintained under a 14 h light–10 h dark photoperiod in synthetic 2.1 ppt water made with Instant Ocean^®^ salt (Unified Pet Group, Inc., Blacksburg, VA, USA) and deioinized water. After 10–15 days in the 208 litre holding tanks, pupfish were assigned to 38 litre experimental tanks (four replicate tanks per treatment), with four fish (two males and two females) per tank. Fish from each population were maintained separately in these 38 litre tanks for 88 days in two temperature conditions: 24 or 34°C. Temperature measurements recorded using HOBO^®^ U12 External Data Loggers (Onset Corp., Bourne, MA, USA) over the 88 days period confirmed the temperature treatments as 23.97 ± 0.40 and 34.18 ± 0.24°C (means ± SD). On the day immediately prior to sacrifice, four tanks from each population–temperature combination were treated with waterborne T_3_ (dissolved in 0.01 M NaOH; Sigma-Aldrich) for an exposure dose of 15 nM (for similar methods, see Johnson and Lema, 2011; Jones *et al*., 2016). All other tanks received control vehicle only. At 18–24 h after hormone treatment, pupfish (*n* = 120) were netted from the experimental tanks, euthanized (MS222), and weighed and measured. Blood was centrifuged (850 × g for 10 min at 4°C) for plasma collection, and liver and skeletal muscle tissues were dissected, flash frozen in liquid N_2_ and stored at −80°C. All fish were sampled between the hours of 13.00 and 16.00 h to reduce photoperiod effects.

### Radioimmunoassays of T_4_ and T_3_

Plasma total T_4_ and total T_3_ were quantified using radioimmunoassay (RIA). Detailed methods for this RIA procedure are provided by Dickhoff *et al*. (1982). Anti-L-T_4_ antiserum (1:3500) and anti-L-T_3_ antiserum (1:10 000) were obtained from Fitzgerald Industries International (Acton, MA, USA). Given the small body size of *C. n. amargosae* [typically <40 mm standard length (SL)], plasma from some fish was pooled to obtain sufficient volumes for RIA. Samples were run in duplicate when allowed by plasma volume. The intra-assay percentage coefficient of variation for the T_3_ RIA was 4.83%, and the inter-assay coefficient of variation was 6.21%. All samples for the T_4_ RIA were run singly because of limited plasma volume.

Pupfish acclimated to 24 or 34°C for 88 days and then treated with exogenous T_3_ were confirmed to have elevated plasma T_3_ concentrations, with the magnitude of that elevation influenced by acclimation temperature (temperature × hormone: *F*_1,61_ = 8.732, *P =* 0.0044; Table 1). Mean plasma T_3_ ranged between 11.3 and 15.1 ng/ml for pupfish not treated with exogenous T_3_, and there was no apparent effect of population origin or temperature acclimation on T_3_ concentrations in those fish. Plasma T_3_ increased to 95.0 ng/ml (mean, both populations combined) in pupfish at 24°C after 18–24 h of exposure to waterborne T_3_ (15 nM), but to 328.4 ng/ml in fish acclimated to 34°C, indicating that temperature strongly influenced the increase in circulating T_3_ following exogenous T_3_ treatment.

**Table 1:** Plasma 3,5,3′-triiodothryonine (T_3_; mean ± SEM) in pupfish acclimated to 24 or 34°C and treated with waterborne T_3_ (15 nM)

Population	Acclimation temperature	Hormone treatment	Plasma T_3_ (ng/ml)
Amargosa River	24°C	Control	11.31 ± 2.03^a^
		T_3_	99.26 ± 8.87^b,c^
	34°C	Control	12.73 ± 2.02^a^
		T_3_	308.49 ± 76.23^c,d^
Tecopa Bore	24°C	Control	15.08 ± 5.38^a^
		T_3_	89.69 ± 24.95^b^
	34°C	Control	12.72 ± 1.29^a^
		T_3_	343.97 ± 36.85^d^

Superscript letters indicate significantly different plasma hormone concentrations (Tukey's HSD comparisons among all treatment groups; α = 0.05).

### Citrate synthase and lactate dehydrogenase enzyme activity

Enzyme activity for both CS and LDH was quantified using methods described by Walsh and Henry (1990), with modifications to the CS assay as presented by Boyle *et al*. (2003) and Johnson *et al*. (2004). Liver and skeletal muscle tissues were homogenized (Fisher Scientific PowerGen Homogenizer) in 5–65 volumes of Tris–HCl buffer (50 mM Tris–HCl, 1 mM EDTA, 2 mM MgCl_2_ and 2 mM dithiothreitol, pH 7.6) then centrifuged at 16 000***g*** for 20 min at 4°C. Supernatants were removed and stored at −80°C until assayed.

For CS activity, 50 μl of supernatant was added to 1.9 ml of Tris–HCl buffer (50 mM Tris–HCl and 0.1 mM 5,5-dithio-bis-(2-nitrobenzoic acid) [DTNB or Ellman's reagent], pH. 8.1) containing 0.1 mM acetyl CoA and allowed to rest for 5 min to achieve a stable baseline absorbance. The reaction was initiated by the addition of 50 μl of 20 mM oxaloacetate. We recorded the increase in absorbance at 412 nm (P300 Nanophotometer) over 5 min.

To quantify LDH activity, a 10 μl sample of 1:10 diluted supernatant (in 50 mM Tris–HCl buffer, pH 7.6) was added to 1.94 ml of Tris–HCl buffer (50 mM Tris–HCl, pH 7.6) containing 0.12 mM NADH. The reaction was initiated by the addition of 50 μl of 80 mM pyruvate, and the decrease in absorbance at 340 nm was recorded.

The CS and LDH activity (in micromoles per minute per gram of tissue) was calculated using the slope of the absorbance change immediately after addition of the oxaloacetate or pyruvate. We used a molar absorption coefficient of 14.15 ml μmol^−1^ cm^−1^ for reduced DTNB at 412 nm, and 6.22 ml μmol^−1^ cm^−1^ for NADH at 340 nm. All assays were performed in 1.5 ml methacrylate cuvettes at 25°C.

### Quantitative real-time reverse transcriptase–polymerase chain reaction

Quantitative real-time RT-PCR (qRT-PCR) was performed in accordance with the guidelines of Bustin *et al*. (2009). Measured transcripts linked to TH physiology included deiodinases *dio1*, *dio2* and *dio3*, TH receptors *trαA*, *trαB* and *trβ* and the TH membrane transporters *mct8* (solute carrier family 16, member 2; slc16a2) and *oatp1c1* (slco1c1). Partial cDNAs encoding *dio1* (**KT879790**), *dio2* (**KT879791**) and *dio3* (**KT897792**) and TH receptors *trαA* (**KT879793**), *trαB* (**KT879794**) and *trβ* (**KT879795**) were isolated and sequenced from *C. n. amargosae* for the design of gene-specific primers for SYBR Green qRT-PCR (see Supplementary material). Protein coding regions for *mct8* (located within accession no. **JSUU01001843**; http://www.ncbi.nlm.nih.gov/Traces/wgs/) and *oatp1c1* (**JSUU01029502**) were identified from an unannotated genome of the closely related pupfish *C. n. pectoralis* (GenBank accession no. **GCA_000776015**) that became available while this study was ongoing.

We also used qRT-PCR to quantify relative mRNA levels of several metabolism-associated genes, including the mitochondrial ATPase6/8 (*atpase6/8*) and cytochrome oxidase subunit-2 (*cox2*), and the nuclear genes citrate synthase (*cs*), l-lactate dehydrogenase A chain (*ldhA*), B chain (*ldhB*) and C chain (*ldhC*), cytochrome *c* oxidase subunit-5b (*cox5b*), nuclear respiratory factor 1 (*nrf1*) and peroxisome proliferator-activated receptors α (*pparα*) and *δ* (*pparδ*). In addition, we quantified heptic mRNA levels for the orphan nuclear receptors estrogen-related receptor α (*esrra*) and estrogen-related receptor γ (*esrrg*), which regulate mitochondrial gene expression and biogenesis, and gluconeogenesis, respectively (Villena *et al*., 2007; Kim *et al*., 2012). SYBR Green primers for *atpase6/8* and *cox2* were designed using the complete mitogenomes of the Death Valley pupfishes *C. n. amargosae* (**KU883631**) and *C. diabolis* (**KX061747**; Lema *et al*., 2016), and primers for transcripts encoding all other proteins were designed to coding region sequences obtained from the *C. n. pectoralis* genome (**GCA_000776015**). When possible, primers were designed to span an intron boundary. All primers were synthesized by Eurofins MWG Operon (Huntsville, AL, USA). Primer sequences and corresponding Genbank accession numbers are provided in Supplementary material Table S1. The specificity of each primer set was confirmed by cloning (TOPO^®^ TA Cloning, Life Technologies) and Sanger sequencing select PCR products (Molecular Cloning Laboratories, South San Francisco, CA, USA).

For all tissues, total RNA was extracted using Tri-Reagent (Molecular Research Center, Inc.) with bromochloropropane as the phase separation reagent. The resulting RNA was DNase I treated (TURBO DNA-free Kit; Ambion) and quantified using a P300 NanoPhotometer (Implen, Inc.; 260:280 ratios >1.96). For the evaluation of mRNA levels in wild pupfish, total RNA from the liver was reverse transcribed in 16 μl reaction volumes containing 8 μl of total RNA (15 ng/μl), 0.8 μl dNTPs (Promega), 0.8 μl random primers (500 μg/ml; Promega), 0.0625 μl recombinant RNasin^®^ ribonuclease inhibitor (40 u/μl; Promega), 0.1375 μl nuclease-free H_2_O, and 3.2 μl 5× buffer, 2.4 μl MgCl_2_ and 0.6 μl GoScript™ reverse transcriptase (Promega) using a thermal profile of 25°C for 5 min and 42°C for 1 h, followed by 70°C for 15 min to inactivate the reverse transcriptase. For examination of relative mRNA levels in the liver and muscle of pupfish acclimated to conditions of 24 or 34°C, reverse transcription reactions were run with identical reagent composition as described above but scaled proportionally for a 45 μl reaction volume.

All qRT-PCR reactions were conducted at 16 μl, containing 8.0 μl iQ Universal SYBR Green Supermix (Bio-Rad, Hercules, CA, USA), 0.8 μl each of forward and reverse primers (10 μM), 4.9 μl nuclease-free water (Sigma, St Louis, MO, USA) and 1.5 μl of reverse-transcribed cDNA template. Assays were run on a 7300 Real-Time PCR System (Applied Biosystems, Inc.) using a thermal profile of 50°C for 2 min, 95°C for 10 min, and 42 cycles of 95°C for 15 s and 59°C for 1 min, followed by a melt curve analysis.

Standard curves were made for each tissue from a pool of RNA from samples representing all factor categories (e.g. population, temperature, hormone treatment) and sexes. Each standard was serially diluted and assayed in triplicate. DNA contamination was assessed by analysing RNA samples that were not reverse transcribed. Each qPCR run also included samples without cDNA as a further control. The PCR efficiencies for each gene were calculated as percentage efficiency = [10(^1/slope^) − 1] × 100, and are provided in Supplementary material Table S1. Correlation coefficients (*r*^2^) were >0.98 for the standard curve for all genes. Relative expression levels of elongation factor 1α (**EU906930**; Lema *et al*., 2010) and 60S ribosomal protein L8 (*rpl8*; **KJ719257**; Lema *et al*., 2015) were quantified as internal control genes. In the liver of wild pupfish, only *rpl8* mRNA levels were stable across populations and sexes, and data were normalized to *rpl8* from the same tissue prior to being expressed as a relative level. For pupfish acclimated to 24 or 34°C in captivity, relative expression levels of each gene were normalized to the geometric mean of *ef-1α* and *rpl8* mRNA levels, which did not vary with population, temperature and hormone treatment.

### Statistical analyses

Relative gene transcript abundance data from the liver of wild pupfish were log_10_(*x* + 1) or sqrt(*x* + 0.5) transformed to conform to parametric assumptions. Population differences in plasma T_4_ and mRNA abundance were assessed using Student's unpaired *t*-tests (α = 0.05).

Plasma T_3_ concentrations in captive-held fish were log_10_ transformed and compared using a three-factor ANOVA model with population origin, temperature condition and hormone treatment as factors. Tukey's HSD tests were then run to determine pairwise differences. Relative gene transcript abundance data from the liver and skeletal muscle were log_10_(*x* + 1) transformed to meet parametric assumptions when needed. Prior studies have found sex differences in TH signalling-associated gene expression in the liver of fish (e.g. Lema *et al*., 2009; Johnson and Lema, 2011); for that reason, sex was included as a factor in the statistical analyses. Four-factor ANOVA models with population origin, temperature condition, hormone treatment and sex as factors and all interactions were used to examine sources of variation for the expression level of each gene transcript. For liver deiodinase mRNA levels, planned pairwise comparisons were calculated to test for temperature or T_3_ exposure effects within the same sex and population only. As sex differences were rarely observed for all other TH signalling- and metabolism-associated transcripts, planned comparisons for those mRNAs were calculated using data from both sexes combined. To reduce type I errors, only statistical differences at α = 0.025 are reported from these pairwise comparisons, and categorical levels of statistical significance are provided to allow evaluation of the relative importance of each effector.

Metabolic enzyme activity data were likewise log_10_(*x* + 1) transformed and then analysed using ANCOVA models with population origin, temperature condition, hormone treatment and sex as main effect factors, and body size (SL, in millimetres) as a covariate. The ANCOVA models included all interaction terms. Outliers in the data were defined as values greater than four standard deviations from the mean of that individual's treatment group. Such outliers included two individuals for lactate dehydrogenase activity in the liver and a single individual for citrate synthase in the liver; those outliers were excluded from ANCOVAs. Only planned pairwise comparisons (*t*-tests) compliant to a significance value of α = 0.025 are reported. All tests were two tailed, and data are plotted as mean ± SEM values. All analyses were completed using JMP^®^ 11.2.0 software (SAS Institute, Inc.).

## Results

### Thyroid hormone signalling variation between wild populations

Plasma T_4_ concentrations were similar in adults from the two wild populations [*P* = 0.629; 19.7 ± 3.1 ng/ml (mean ± SEM) in Amargosa River pupfish and 22.9 ± 5.5 ng/ml in Tecopa Bore pupfish]. Replication after pooling plasma samples from individuals with low volumes was insufficient for sex-specific comparisons between populations.

**Figure 1:**
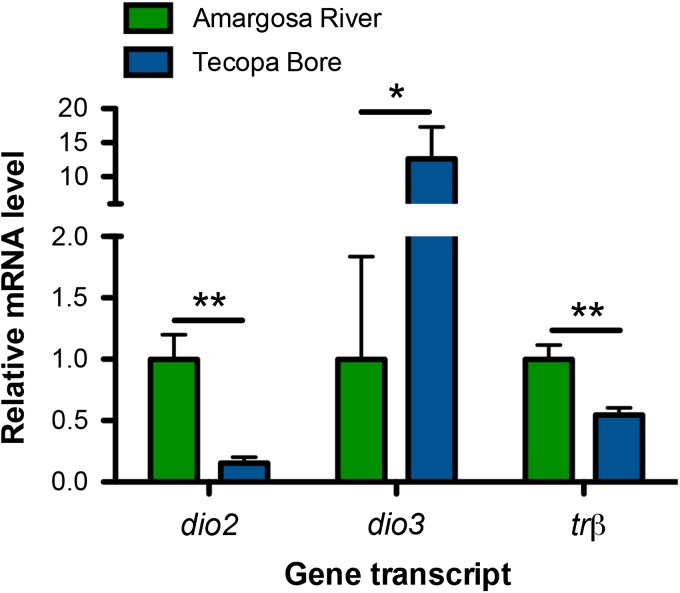
Variation in liver mRNA abundance in wild male pupfish. Males from the wild Amargosa River and Tecopa Bore populations differ in hepatic mRNA abundance for iodothyroinine deiodinase enzymes type 2 (*dio2*) and type 3 (*dio3*), as well as thyroid hormone receptor β (*trβ*). Asterisks indicate significant difference between populations (Student's unpaired *t*-tests: *P < 0.05, **P < 0.01).

Male pupfish from the Amargosa River and Tecopa Bore differed in relative mRNA levels for the deiodinase enzymes *dio2* (*t* = −4.580, *P* = 0.0004, d.f. = 14) and *dio3* (*t* = 2.529, *P* = 0.0241, d.f. = 14). Transcripts encoding *dio2* were >6-fold greater in relative abundance in the liver of males from the Amargosa River, whereas *dio3* mRNAs were 12-fold more abundant in males from Tecopa Bore (Fig. 1). Hepatic *trβ* transcript levels were also ~2-fold higher in male Amargosa River pupfish (*t* = −3.023, *P* = 0.0091, d.f. = 14; Fig. 1). Female pupfish from the two populations did not differ in hepatic *dio2*, *dio3* or *trβ* mRNA abundance. Hepatic *dio1*, *trαA* and *trαB* mRNA levels also did not vary between populations for either sex.

### Temperature and T_3_ influence hepatic gene transcript abundance

**Figure 2:**
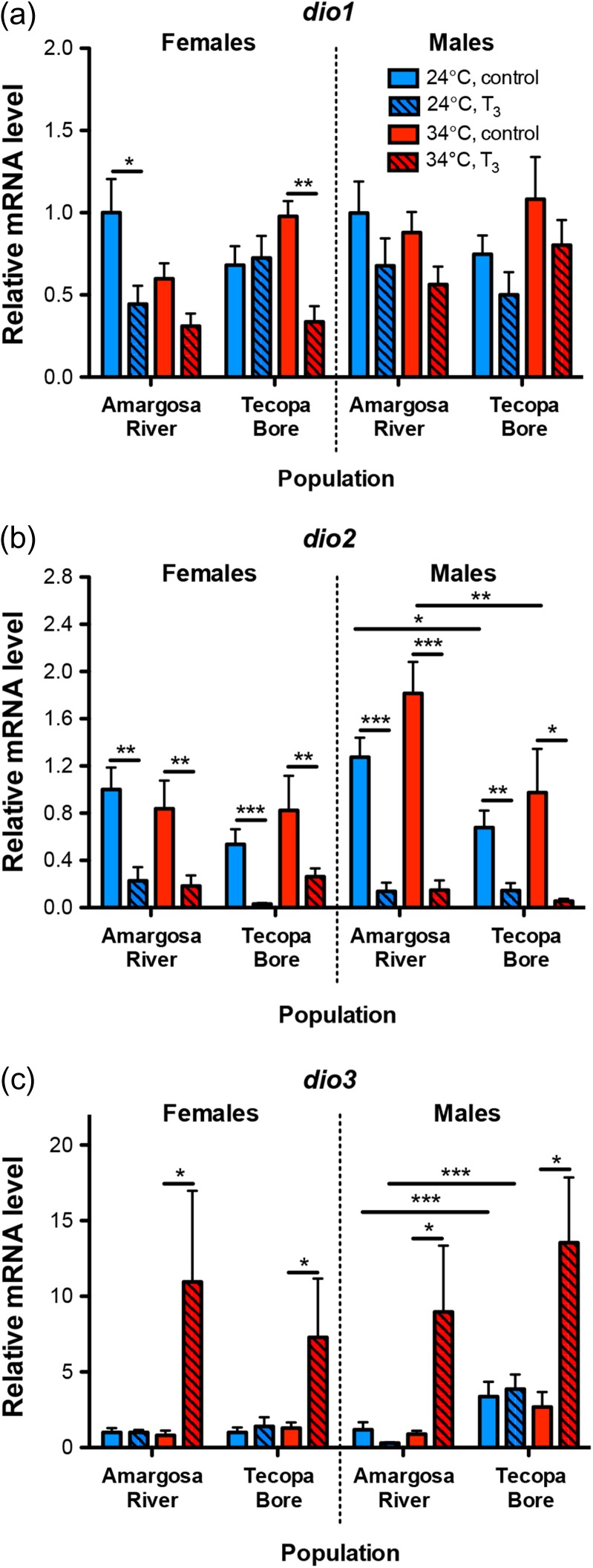
Variation in hepatic deiodinase gene transcript abundance. (**a**) Treatment of fish with 3,5,3′-triiodothryonine (T_3_) reduced the relative level of mRNAs encoding *dio1* in liver (ANOVA model, *P <* 0.0001), although this effect of T_3_ was apparent only in females in pairwise comparisons (α = 0.025). (**b**) 3,5,3′-Triiodothryonine depressed liver *dio2* mRNA abundance in males and females from both populations. Male pupfish from the Amargosa River also had higher *dio2* mRNA levels than males from Tecopa Bore. (**c**) 3,5,3′-Triiodothryonine-induced elevation of hepatic *dio3* transcripts was temperature dependent. In both sexes, T_3_ treatment increased *dio3* mRNAs at 34 but not at 24°C. Male pupfish from Tecopa Bore not treated with T_3_ also had higher hepatic *dio3* mRNA levels compared with Amargosa River males. Lines denote significant pairwise differences between groups (planned Student's unpaired *t*-tests: *P < 0.025, **P < 0.01, ***P < 0.001); *n* = 6–10 fish per category.

The relative abundance of mRNAs encoding deiodinase enzymes in the liver was altered by exogenous T_3_, but the magnitude and direction of those effects varied with population origin and thermal experience. Transcripts encoding *dio1* in the liver were lower in T_3_-treated fish (Fig. 2a; hormone: *F*_1,104_ = 19.228, *P <* 0.0001). Significant declines in hepatic *dio2* transcript abundance were also observed in T_3_-treated males and females from both populations (Fig. 2b). In addition to these T_3_-induced changes, males from the Amargosa River population acclimated to both 24 and 34°C exhibited higher hepatic *dio2* mRNA levels compared with males from Tecopa Bore (population × sex × hormone: *F*_1,104_ = 5.524, *P =* 0.0207). This population difference in liver *dio2* mRNAs was observed in males but not in females. Males from Tecopa Bore had higher hepatic *dio3* transcripts compared with males from the Amargosa River, and T_3_ increased hepatic *dio3* mRNAs >13.5-fold in both populations at 34 but not at 24°C (Fig. 2c; population × temperature × hormone: *F*_1,104_ = 5.592, *P =* 0.0199).

**Figure 3:**
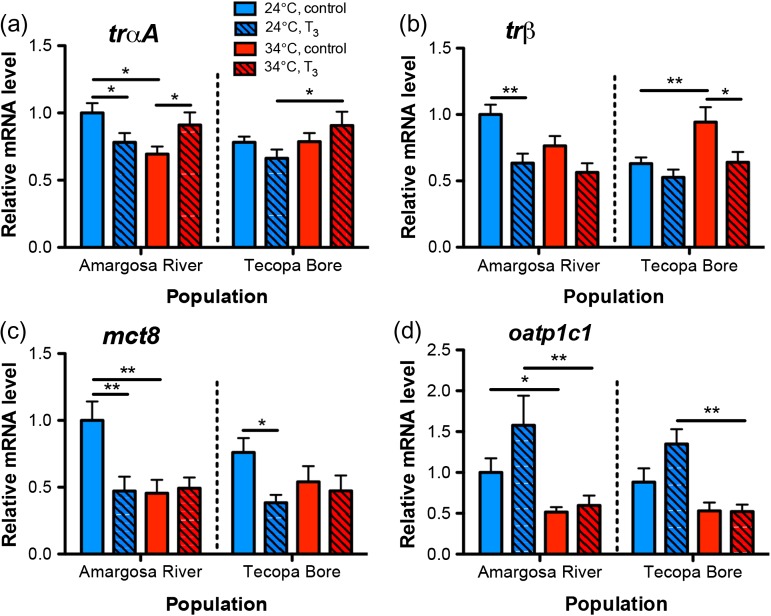
Influences of population, temperature and T_3_ on hepatic thyroid hormone (TH) receptors and membrane transporters. (**a**) 3,5,3′-Triiodothryonine reduced the abundance of liver mRNAs encoding TH receptors *trαA* at 24°C, but increased *trαA* mRNAs at 34°C. (**b**) Liver *trβ* mRNA levels were affected by temperature in opposing directions, depending on population origin, and were also reduced in fish treated with T_3_. Transcript abundance for the TH plasma membrane transporters *mct8* (**c**) and *oatp1c1* (**d**) was reduced in fish at 34 compared with 24°C. 3,5,3′-Triiodothryonine reduced liver *mct8* mRNA levels in the Amargosa River population, but not the Tecopa Bore population, at 24 but not 34°C. Data from males and females are shown combined. Sample sizes are *n =* 14–17 fish per group. Lines indicate significant pairwise differences between groups (planned Student's unpaired. *t*-tests: *P < 0.025, **P < 0.01).

3,5,3′-Triiodothryonine altered hepatic *trαA* mRNA levels (Fig. 3a), although the direction of this T_3_ effect varied depending on acclimation temperature (temperature × hormone: *F*_1,104_ = 13.664, *P =* 0.0004). In both populations, T_3_ induced an increase in hepatic *trαA* transcripts at 34°C, but reduced *trαA* mRNA abundance at 24°C. Amargosa River pupfish not treated with T_3_ exhibited higher hepatic *trαA* mRNA levels at 24 than at 34°C, even though *trαA* transcript abundance in Tecopa Bore pupfish did not vary with temperature (population × temperature: *F*_1,104_ = 6.217, *P =* 0.0142). Males also exhibited greater levels of hepatic *trαA* mRNAs compared with females (sex: *F*_1,104_ = 18.903, *P <* 0.0001; result not shown). Patterns of variation in liver *trβ* mRNA abundance followed a distinct pattern from the variation observed for *trαA*. Amargosa River fish at 24°C had higher liver *trβ* mRNA levels than those at 34°C, whereas Tecopa Bore pupfish showed the opposite pattern, with more abundant *trβ* transcripts at 34 compared with 24°C (Fig. 3b; population × temperature: *F*_1,104_ = 14.713, *P =* 0.0002). Liver *trβ* transcript abundance was reduced by T_3_ in both populations and at both temperatures (hormone: *F*_1,104_ = 20.348, *P* < 0.0001). In both populations, males had higher hepatic *trβ* mRNA levels than females (sex: *F*_1,104_ = 8.579, *P =* 0.0042; result not shown). Liver *trαB* mRNA abundance was not affected by population origin, sex, temperature or T_3_.

Treatment with T_3_ reduced hepatic *mct8* mRNAs in pupfish acclimated to 24 but not to 34°C (Fig. 3c; temperature × hormone: *F*_1,97_ = 7.537, *P =* 0.0072). Male pupfish had ~10% higher liver *mct8* mRNA levels than females, although the size of this sex difference varied with population origin and acclimation temperature (population × temperature × sex interaction: *F*_1,97_ =11.421, *P =* 0.0010). Transcripts encoding *oatp1c1* were less abundant in fish acclimated to 34 compared with 24°C (Fig. 3d; temperature: *F*_1,104_ = 35.395, *P <* 0.0001) and showed a small elevation in expression in T_3_-treated fish at 24°C (hormone: *F*_1,104_ = 4.211, *P =* 0.0427).

### Effects on muscle gene transcript abundance

**Figure 4:**
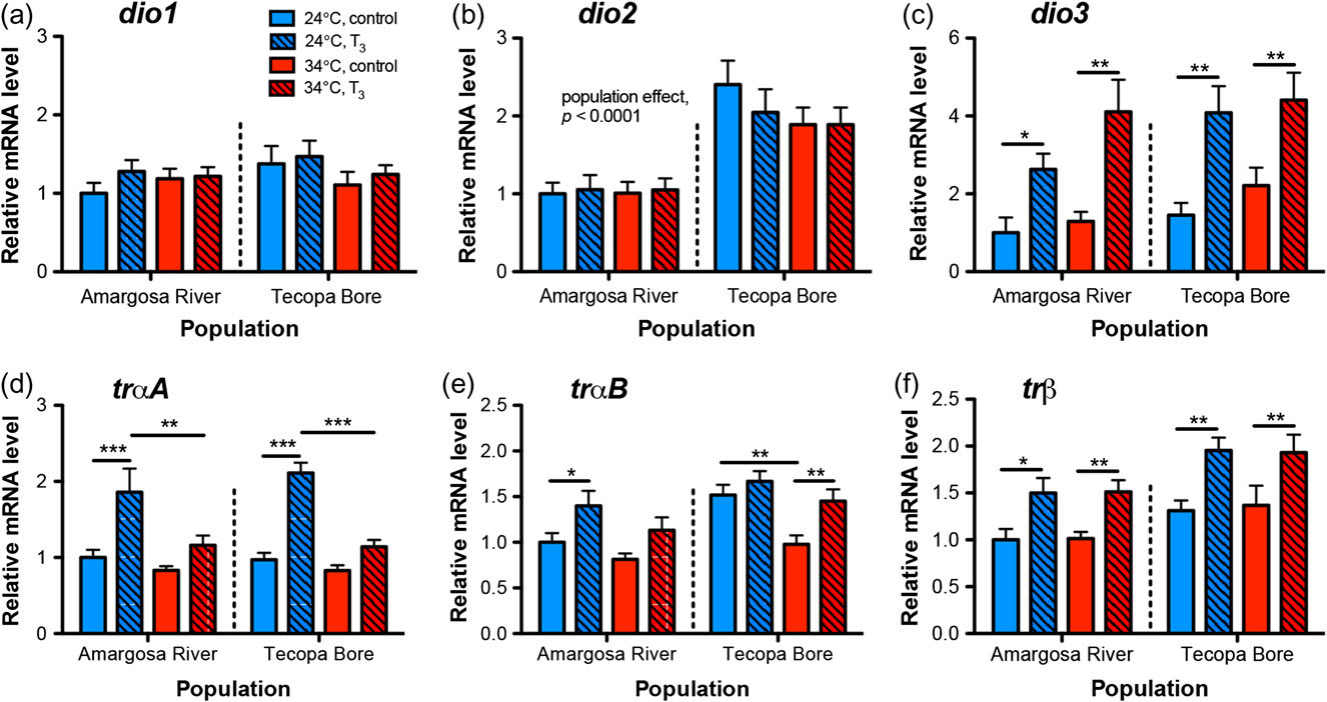
Iodothyronine deiodinase enzyme and TH receptor mRNA levels in skeletal muscle. (**a**) Muscle deioinase type 1 (*dio1*) mRNAs did not vary with population origin, thermal experience or T_3_ supplementation. (**b**) Muscle *dio2* transcript abundance was ~2-fold greater in Tecopa Bore pupfish than in Amargosa River pupfish, but was not altered by temperature or T_3_. (**c**) Muscle *dio3* mRNA levels were up-regulated by T_3_. (**d**) Thyroid hormone receptor *trαA* mRNA levels were up-regulated ~2-fold by T_3_ at 24°C, but only ~0.2-fold at 34°C. (**e**) TH receptor *trαB* mRNA levels in muscle were down-regulated at high temperature in the absence of supplemental T_3_, and increased in relative abundance with T_3_ treatment. (**f**) Muscle *trβ* mRNAs were increased in the Tecopa Bore population compared with Amargosa River fish and were up-regulated in both populations by T_3_. Data from males and females are shown combined. Sample sizes are *n =* 14–17 fish per group. Lines indicate significant differences (planned Student's unpaired *t*-tests: *P < 0.025, **P < 0.01, ***P < 0.001).

The relative abundance of *dio2* mRNAs in skeletal muscle was higher in pupfish from Tecopa Bore than in conspecifics from the Amargosa River (Fig. 4b; population: *F*_1,103_ = 27.233, *P <* 0.0001). Transcript abundance for *dio2* was not affected by either temperature or T_3_ in either population. Muscle *dio3* mRNA levels were higher in T_3_-treated fish (Fig. 4c; hormone: *F*_1,103_ = 38.709, *P <* 0.0001). Both populations also exhibited greater muscle *dio3* transcript abundance at 34 compared with 24°C (temperature: *F*_1,103_ = 4.838, *P =* 0.0301). Relative deiodinase *dio1* transcript abundance was not affected by temperature, population origin or hormone treatment (Fig. 4a).

3,5,3′-Triiodothryonine increased the relative abundance of transcripts encoding *trαA* in both populations and sexes at 24 but not at 34°C (Fig. 4d; temperature × hormone: *F*_1,103_ = 11.605, *P =* 0.0009). 3,5,3′-Triiodothryonine likewise induced an increase in muscle *trαB* transcript abundance (hormone: *F*_1,103_ = 15,273, *P =* 0.0002). Muscle *trαB* mRNAs were also higher in Tecopa Bore pupfish compared with Amargosa River pupfish (Fig. 4e; population: *F*_1,103_ = 14.145, *P =* 0.0003) and were more abundant at 24 than at 34°C in both populations (temperature: *F*_1,103_ = 12,154, *P =* 0.0007). Muscle *trβ* mRNAs in both populations and at both acclimation temperatures were elevated ~45% by T_3_ treatment (Fig. 4f; hormone: *F*_1,103_ = 31.972, *P <* 0.0001) and were also higher at both acclimation temperatures in Tecopa Bore pupfish compared with the Amargosa River population (population: *F*_1,103_ = 13.787, *P =* 0.0003). Transcripts for *mct8* and *oatp1c1* were not quantified in skeletal muscle because prior studies in fish indicate very low mRNA abundance for these solute carriers in this tissue (Muzzio *et al*., 2014).

### Metabolic enzyme activity in skeletal muscle

**Figure 5:**
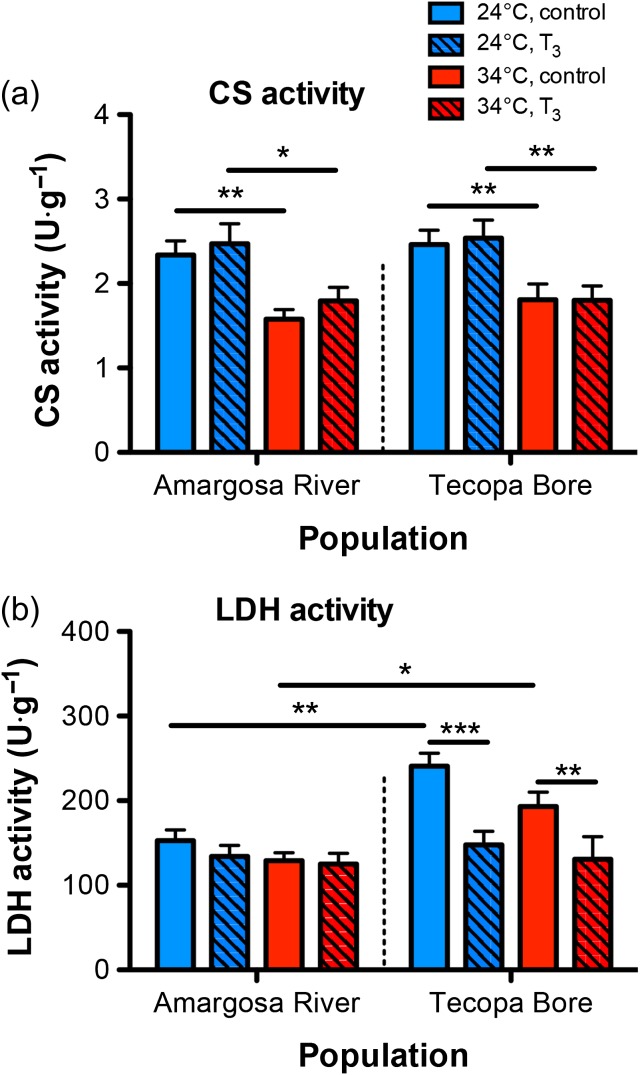
Citrate synthase (CS) and lactate dehydrogenase (LDH) activity in skeletal muscle. (**a**) Muscle aerobic CS activity was reduced in fish acclimated to 34°C and was unaffected by T_3_ at either acclimation temperature. (**b**) Lactate dehydrogenase activity in muscle was higher in pupfish from the Tecopa Bore population compared with fish from the Amargosa River. 3,5,3′-Triiodothryonine reduced LDH activity in Tecopa Bore pupfish acclimated to both 24 and 34°C but had no effect in Amargosa River pupfish at either temperature. Sample sizes are *n =* 6–10 fish per group. Lines indicate significant differences (planned Student's unpaired *t*-tests: *P <* 0.025, **P < 0.01, ***P < 0.001).

The activity of CS in muscle was reduced at 34 compared with 24°C (Fig. 5a; population × temperature × SL interaction: *F*_1,101_ = 5.850, *P =* 0.0174). Supplementation with T_3_ had no influence on CS activity at either temperature. Exogenous T_3_, however, depressed muscle LDH activity in pupfish from Tecopa Bore but not from the Amargosa River (Fig. 5b; population × hormone: *F*_1,101_ = 5.101, *P =* 0.0261). Hepatic CS and LDH activity is provided in the Supplementary material (Fig. S1).

### Metabolic gene expression in skeletal muscle

**Figure 6:**
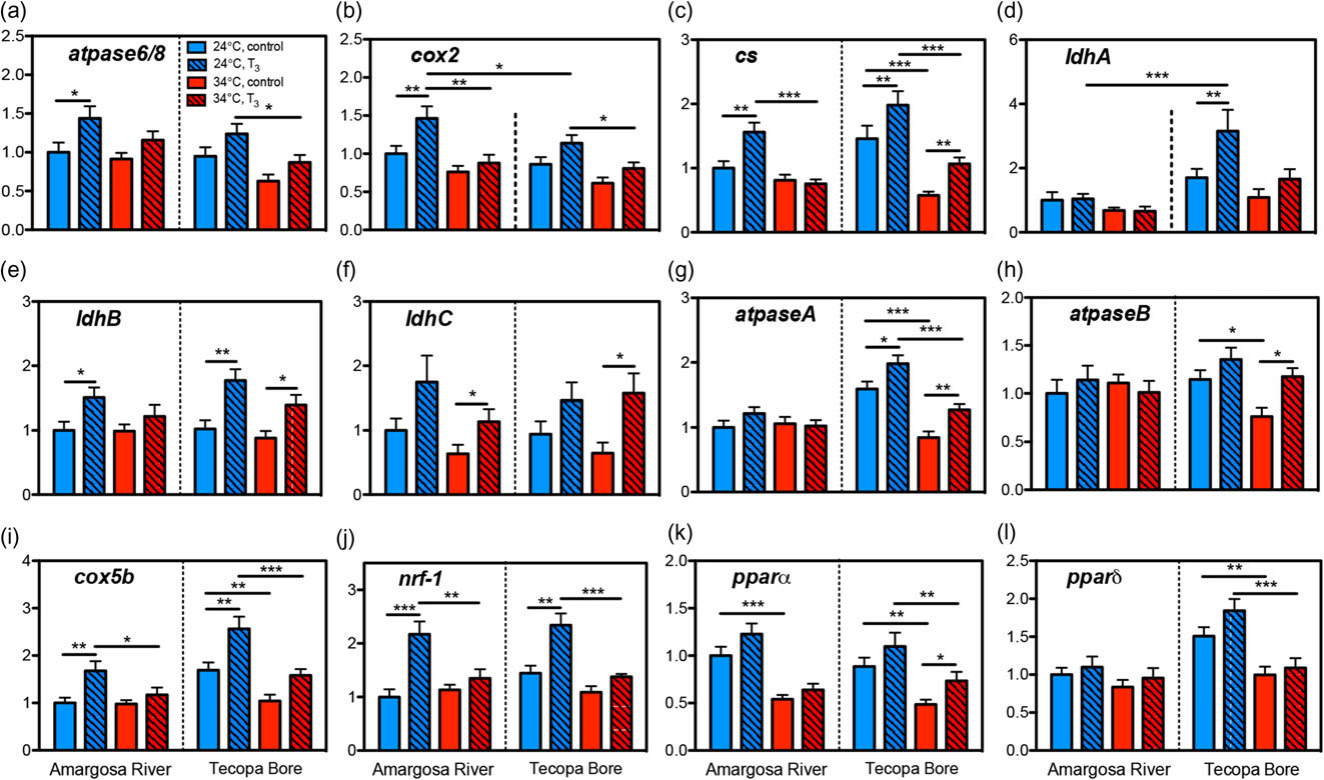
Metabolism-associated gene transcripts in skeletal muscle. Relative mRNA levels for mitochondrial *atpase6/8* (**a**), mitochondrial *cox2* (**b**), *cs* (**c**), *ldhA* (**d**), *ldhB* (**e**), *ldhC* (**f**), *atpaseA* (**g**), *atpaseB* (**h**), *cox5b* (**i**), *nrf-1* (**j**), *pparα* (**k**) and *pparδ* (**l**). Note that scales of *y*-axis relative mRNA levels differ among transcripts. Sample sizes are *n =* 14–17 fish per group. Lines indicate pairwise differences between groups (planned Student's unpaired *t-*tests: *P < 0.025, **P < 0.01, ***P < 0.001).

Transcripts encoding the mitochondrial genes *atpase6/8* and *coxII* were at higher relative abundance in the Amargosa River population compared with Tecopa Bore (Fig. 6a and b; population: *atpase6/8*, *F*_1,103_ = 4.766, *P =* 0.0313; *coxII*, *F*_1,103_ = 5.745, *P =* 0.0183). Both mitogenome transcripts were also increased by T_3_ treatment (*atpase6/8*, *F*_1,103_ = 9.647, *P =* 0.0025; *coxII*, *F*_1,103_ = 14.095, *P =* 0.0003) and at reduced relative levels at 34 compared with 24°C (*atpase6/8*, *F*_1,103_ = 6.473, *P =* 0.0124; *coxII*, *F*_1,103_ = 21.145, *P <* 0.0001). Relative *cs* mRNA levels in muscle increased in response to T_3_ (Fig. 6c; hormone: *F*_1,103_ = 15.658, *P =* 0.0001). Transcript abundance for *cs* was lower in Tecopa Bore fish at 34 compared with 24°C; however, this temperature effect was not observed in the Amargosa River population (population × temperature: *F*_1,103_ = 5.237, *P =* 0.0242). The relative expression of transcripts encoding the LDH A-chain gene *ldhA*, but not *ldhB* or *ldhC*, differed between populations. Messenger RNA levels for *ldhA* were higher in Tecopa Bore pupfish than in Amargosa River fish, and also exhibited a T_3_-induced increase in Tecopa Bore fish only (Fig 6d; population × hormone: *F*_1,103_ = 4.796, *P =* 0.0308). Fish from both populations had lower *ldhA* mRNA levels at 34 than at 24°C (*F*_1,103_ = 12.663, *P =* 0.0006). Transcripts for *ldhB* and *ldhC* were increased in T_3_-treated fish (Fig. 6e and f), although (unlike for *ldhA*) this hormone effect occurred in both populations (*ldhB*, *F*_1,103_ = 6.921, *P =* 0.0098; *lhdC*, *F*_1,103_ = 14.631, *P* = 0.0002).

Several other metabolism-associated genes were lower in the skeletal muscle of pupfish acclimated to 34°C. Transcripts for *atpaseA* were more abundant at 24 compared with 34°C in pupfish from Tecopa Bore but not from the Amargosa River (Fig. 6g; population × temperature: *F*_1,103_ = 18.531, *P <* 0.0001). 3,5,3′-Triiodothryonine increased both *atpaseA* (population × hormone: *F*_1,103_ = 4.752, *P =* 0.0315) and *atpaseB* (Fig. 6h; population × hormone: *F*_1,103_ = 4.690, *P =* 0.0326) mRNA levels, although again this effect was observed in the Tecopa Bore population only. 3,5,3′-Triiodothryonine increased *cox5b* mRNA levels in both populations and temperature conditions (Fig. 6i; hormone: *F*_1,103_ = 27.245, *P <* 0.0001). Tecopa Bore pupfish also expressed lower relative *cox5b* mRNA levels in muscle at 34 than at 24°C, but this temperature effect was not observed in the Amargosa River population (population × temperature: *F*_1,103_ = 5.847, *P =* 0.0174). Transcripts encoding *nrf-1* were increased by T_3_ at 24 but not at 34°C (Fig. 6j; temperature × hormone: *F*_1,103_ = 11.197, *P =* 0.0011). Muscle *pparα* mRNAs were reduced at 34 compared with 24°C (Fig. 6k; temperature: *F*_1,102_ = 49.827, *P <* 0.0001). Transcripts encoding *pparδ* were likewise lower in Tecopa Bore pupfish—but not Amargosa River pupfish—acclimated to 34 compared with 24°C (Fig. 6l; population × temperature: *F*_1,103_ = 10.354, *P =* 0.0017). 3,5,3′-Triiodothryonine increased the abundance of mRNAs for *pparα* (hormone: *F*_1,102_ = 9.955, *P =* 0.0022) and *pparδ* (hormone: *F*_1,103_ = 5.624, *P =* 0.0196) in muscle irrespective of population origin or temperature. Data for mRNA expression of metabolism-associated genes in the liver are provided in the Supplementary material (Figs S2 and S3).

## Discussion

Many native fishes of North America's southwestern deserts have protected ‘endangered’ status, and the small population sizes of these species continue to make them vulnerable to a variety of anthropogenic impacts (Minckley *et al*., 1991; Moyle *et al*., 2011). Climate models predict that increasingly warm and arid conditions in the North American southwest will intensify the region's already extreme temperatures (Seager *et al*., 2007; Seager and Vecchi, 2010). Such temperature changes are likely to impact the region's native fishes given that many of these taxa occur only in a few small, isolated habitats and have limited ability to shift their geographical range (e.g. Hausner *et al*., 2014). Assessing patterns of local population variation in thermal physiology will therefore be crucial for identifying populations most likely to persist under a changing climate (Chown *et al*., 2010; Hoffman and Sgrò, 2011).

Here, we provide evidence for variation in TH physiology in two wild, allopatric populations of *C. n. amargosae* pupfish. Specifically, we found that male *C. n. amargosae* pupfish sampled directly from the Amargosa River habitat exhibited 5-fold greater liver *dio2* mRNA levels compared with males in Tecopa Bore, whereas liver *dio3* mRNAs were ~12.5-fold greater in abundance in Tecopa Bore males. These differences in deiodinase mRNA levels were observed without any evidence for variation in circulating T_4_ between the populations, but were paralleled by ~2-fold higher hepatic mRNA levels for the TH receptor *trβ* in Amargosa River males. As temperature can influence TH pathways in fish (Leatherland *et al*., 1977, 1980; Eales *et al*., 1982), and the temperature of Tecopa Bore is, on average, higher than that of the Amargosa River, we acclimated pupfish from these populations to stable conditions of 24 or 34°C to test whether the observed variation in TH pathway-associated mRNA levels between the wild populations arises from dissimilar thermal experience. Although we did not detect any effects of acclimation temperature on endogenous T_3_ concentrations in either population, males from these populations retained differences in liver *dio2* and *dio3* and, to a lesser extent, *trβ* gene transcript abundance even after nearly 3 months of similar thermal experience. Tecopa Bore pupfish also had higher mRNAs levels for *dio2* and *trβ* in skeletal muscle than Amargosa River fish, and this population variation was independent of thermal acclimation.

Dio2 has ORD activity and removes iodine from the 5′ outer-ring site to convert T_4_ to the more active form, T_3_, whereas Dio3 acts as an inner-ring deiodinase and removes iodine from the inner ring of T_4_ and T_3_ to convert these hormones to reverse triiodothyronine (rT_3_) and diiodothyronine (T_2_), respectively (Köhrl, 2000; Orozco and Valverde, 2005; St Germain *et al*., 2009; Little, 2016). The differences in hepatic and muscle *dio2* and *dio3* mRNA levels between the Amargosa River and Tecopa Bore populations therefore implies variation in TH conversion. Given the multiple post-transcriptional regulatory steps between gene transcription and the production of functional proteins, variation in transcript levels does not necessarily translate into differences in enzyme activity (e.g. Maier *et al*., 2009; Ghazalpour *et al*., 2011; Suarez and Moyes, 2012). As such, it is difficult to interpret definitively the functional implications of population-level variation in the *dio* mRNA levels observed between the Amargosa River and Tecopa Bore populations. Even so, recent studies point to TH action being strongly regulated by local variation in the expression of deiodinases and TH transporters at the target tissue (Schweizer *et al*., 2008; Darras *et al*., 2015). If the mRNA abundance variation observed here associates positively with Dio activity, Tecopa Bore pupfish may have increased TH inactivation by Dio3 in the liver, whereas the ~2-fold higher *dio2* mRNA levels in skeletal muscle may indicate elevated conversion of T_4_ to T_3_ in that tissue.

Thyroid hormones mediate changes in gene expression via the action of nuclear TH receptors, which bind to thyroid response elements (TREs) to regulate transcription (Yen *et al*., 2006). A wide variety of genes have been found to be regulated by THs (e.g. Chatonnet *et al*., 2015; Gil-Ibañez *et al*., 2015), although perhaps the best-studied TH targets are genes encoding proteins involved in TH signalling pathways and metabolism. For instance, previous studies in fishes have demonstrated up-regulation of hepatic *dio3* mRNAs and down-regulation of *dio2* mRNAs in response to exogenous T_3_ (García-G *et al*., 2004; Bres *et al*., 2006; Lema *et al*., 2009; Johnson and Lema, 2011). Our data here confirm these general patterns of T_3_ induction of *dio3* and down-regulation of *dio2* in the liver, suggesting that the changes in mRNA levels observed here may correlate with similar variation in Dio activity. Moreover, our data provide evidence for T_3_ regulation of *dio3*, but not *dio2*, in skeletal muscle. Thyroid hormone receptor genes of mammals and amphibians contain TREs and are autoinduced by T_3_ (Yen *et al*., 2006), and indications are that *trαA* (or *trα*) and *trβ* transcription may be autoinduced in teleost fishes as well (Kawakami *et al*., 2006; Nelson and Habibi, 2006; Johnson and Lema, 2011; Nelson *et al*., 2011). Our findings here provide further support for *tr* autoinduction by T_3_, with transcripts encoding *trαA*, *trαB* and *trβ* in muscle detected at higher levels in T_3_-treated pupfish.

To our knowledge, the present findings provide the first evidence in fish for temperature effects on expression of the TH transporters Mct8 and Oapt1c1. The Mct8 and Oatp1c1 solute carriers facilitate TH transport across the cellular plasma membrane (Arjona *et al*., 2011; Visser *et al*., 2011), and changes in expression of these carriers have the potential to influence the transport rates of T_4_ and T_3_ into target cells. 3,5,3′-Triiodothryonine has also been observed to regulate hepatic gene expression of *mct8* and *oatp1c1* in the fathead minnow, *Pimepheles promelas* (Noyes *et al*., 2013; Muzzio *et al*., 2014). In the present study, T_3_ down-regulated liver *mct8* mRNA levels while concurrently up-regulating *oatp1c1* mRNAs in pupfish acclimated to 24°C. In zebrafish, Mct8 transport of THs is temperature dependent, transporting only T_3_ at 26°C, but both T_3_ and T_4_ at 37°C (Arjona *et al*., 2011). Although no data on the transport selectivity of Mct8 or Oatp1c1 are available for other fishes, the temperature-dependent TH selectivity observed by Arjona *et al*. (2011) combined with our finding of temperature effects on *mct8* and *oatp1c1* mRNA levels in pupfish suggests that hepatic T_3_ and T_4_ uptake or release may vary with temperature.

In mammals, THs influence metabolic and thermogenic functions by increasing heart rate, promoting hepatic glycogenolysis and gluconeogenesis, increasing acetyl-CoA consumption, elevating respiratory chain protein activity (e.g. ATP synthase) and increasing glucose absorption by the gut (Yen, 2001). Elevated TH status increases energy expenditure in mammals (Vaitkus *et al*., 2015), and these energetic changes are mediated both via the direct action of THs on nuclear gene expression and through TH induction of mitochondrial biogenesis (Wrutniak-Cabello *et al*., 2001; Weitzel *et al*., 2003; Collin *et al*., 2009; Weitzel and Iwen, 2011; Lombardi *et al*., 2015). The increased number of mitochondria that result from TH stimulation allow for elevated respiration under scenarios of increased demand, such as during heightened behavioural activity or adaptive thermogenic responses to environmental change. Although the metabolic actions of THs are less well established in fishes, recent findings point to THs mediating metabolic thermal acclimation in zebrafish (Little *et al*., 2013). In zebrafish, THs were observed to regulate the activity of CS, COX and LDH and relative mRNA abundance of several metabolic genes (e.g. *atpaseA*, *atpaseB*, *cox2*, *atpase6/*8, *nrf-1*), with fish acclimated to higher temperature (28°C) less sensitive to metabolic regulation by THs than at low temperature (18°C; Little *et al*., 2013).

Pupfish acclimated to 34°C likewise showed altered metabolic enzyme activity and metabolism-associated mRNA levels in skeletal muscle and liver (see Supplementary material) compared with fish at 24°C. Muscle aerobic CS activity and *cs* mRNA levels were lower in pupfish at 34 than at 24°C, suggesting decreased oxidative metabolic capacity at 34°C. In contrast, LDH activity in skeletal muscle was not affected by thermal acclimation but was greater in pupfish from the Tecopa Bore population compared with fish from the Amargosa River population. Interestingly, T_3_ depressed muscle LDH activity in Tecopa Bore fish but not Amargosa River fish, suggesting population differences in the regulation of anaerobic metabolism by TH.

The differential pattern of LDH subunit mRNA expression observed in these populations suggests that these LDH activity differences may be related to variation in LDH isoform expression. Lactate dehydrogenase is a tetramer, with isoforms composed of combinations of three LDH subunits (A-chain, B-chain and C-chain), each of which is encoded by a separate gene (Quattro *et al*., 1993). Subunit composition generates variation in kinetic properties and thermal stability among LDH isoforms (e.g. Place and Powers, 1984; Henry and Ferguson, 1985), and evolutionary changes in LDH—and, in particular, A_4_-LDH, the muscle isoform of LDH in vertebrates—have been linked to taxonomic variation in anaerobic thermal scope in fishes (e.g. Fields *et al*., 2015). Here, we observed that T_3_-mediated mRNA regulation of *ldhA* in skeletal muscle differed between the populations, with T_3_ up-regulating *ldhA* mRNAs in Tecopa Bore pupfish but not in Amargosa River pupfish. 3,5,3′-Triiodothryonine also up-regulated *ldhB* and *ldhC* transcripts, but in a similar pattern in both populations. Thus, the divergent responses of muscle LDH activity to T_3_ that we observed in the Amargosa River and Tecopa Bore populations might be a result of population-level variation in T_3_ regulation of *ldhA* transcription. Tecopa Bore has higher average temperatures and lower dissolved O_2_ (~1.8 mg/l), so that pupfish occupying this habitat are likely to have a greater reliance on anaerobic metabolism. Little *et al*. (2013) observed T_3_-induction—as well as T_2_-inhibition—of muscle LDH activity in zebrafish pharmacologically made hypothyroid by propylthiouracil exposure, suggesting that TH regulation of anaerobic metabolic activity might occur broadly in fishes. At the same time, these same T_3_-treated zebrafish also showed increased mRNA abundance for several mitochondrial protein subunits, including *atpase6/8*, *cox2*, *atpaseB* and *coxv2b*, suggesting that T_3_ might concomitantly enhance LDH activity and aerobic respiration.

The reduced muscle CS activity observed in both populations at 34°C also suggests a reduced reliance on aerobic metabolism by pupfish at elevated temperature. Given that rising temperatures lower water O_2_ content, this decline in CS activity is likely to enable better survival in lower dissolved O_2_ conditions. Why we did not observe a corresponding increase in LDH activity at 34°C is less clear, however, because fish exposed to elevated temperatures often increase anaerobic metabolic activity. Recent findings by Heuton *et al*. (2015) may point to a possible answer, because these authors proposed that pupfish (*C. n. mionectes* × *C. diabolis* hybrids) may not rely heavily on lactate production when acclimated to high temperature (33°C), but instead enter into a state of ‘paradoxical anaerobism’ wherein fish rely on ethanol production. In the face of observations by Heuton *et al*. (2015), future studies should examine how alcohol dehydrogenase activity varies in pupfish acclimated to varying temperature and dissolved O_2_ conditions.

The patterns of mRNA changes that we observed in the skeletal muscle provide additional evidence for T_3_ induction of aerobic metabolic pathways. 3,5,3′-Triiodothryonine up-regulated mRNAs encoding the mitochondrial genes *atpase6/8* and *cox2* in pupfish muscle, suggesting T_3_ induction of mitogenome transcription and mitochrondrial activity in fish, as has been observed in mammals and birds (Pillar and Seitz, 1997; Psarra *et al*., 2006; Collin *et al*., 2009; Psarra and Sekeris, 2013). We also detected T_3_ induction of mRNAs encoding *cox5b*, *nrf-1*, *pparα* and *pparδ* in muscle, implying a general up-regulation of aerobic metabolic gene expression. For instance, T_3_ treatment increased the relative levels of muscle *nrf-1* mRNAs in both pupfish populations, although this effect was more pronounced at 24 than at 34°C. In the rat liver, TH stimulation of mitochondrial biogenesis and respiratory chain activity is mediated, in part, via T_3_ induction of *nrf-1* and *nrf-2* transcription (Rodríquez-Peña *et al*., 2002). Temperature- and T_3_-associated variation in expression of the peroxisome proliferator-activated receptors (PPARs) *pparα* and *pparδ* in pupfish muscle again points to TH-mediated changes in energy homeostasis. The PPARs are ligand-activated transcription factors, and the activation of PPARα and PPARδ has been linked to changes in mitochondrial and peroxisome fatty acid metabolism (Tyagi *et al*., 2011).

It is crucial to point out, however, that the temperature-associated variation in T_3_-induced mRNA expression observed here might alternatively be related to differential circulating T_3_ concentrations in T_3_-treated fish at 34 compared with 24°C, rather than to variation in transcriptional responses to T_3_ at these two temperatures. We used a waterborne T_3_ treatment method instead of an injection, implantation or oral dosing method to avoid effects of acute stress from netting and handling fish and to minimize dosing variation that would likely have occurred if T_3_ was administered orally (e.g. via food) over the short 18–24 h period. The waterborne exposure, however, raised circulating T_3_ several-fold higher than the endogenous concentrations observed in control pupfish, and this pharmacological dose might have induced effects different from what would have occurred with a physiological dose. Additionally, temperature affected the uptake rate of T_3_ from the water, and comparisons of data from the T_3_-treated pupfish at 24 and 34°C should be interpreted cautiously. Although we are unaware of any study in fish that has examined temperature-dependent kinetics of TH uptake from water, temperature can influence rates of chemical uptake from water in fishes (McKim and Erikson, 1991). For instance, waterborne estrogen uptake across the gill is positively associated with oxygen consumption rates (Blewett *et al*., 2013a,b), which increase at elevated temperatures.

Even so, the variation in gene transcript levels observed between the Amargosa River and Tecopa Bore populations in the absence of T_3_ treatment suggests that these populations vary in patterns of gene expression associated with TH pathways and energy metabolism. The Tecopa Bore population of *C. n. amargosae* was founded by individuals from the Amargosa River <50 years ago, so any underlying genetic bases to these TH pathway and metabolic differences could represent contemporary evolution resulting from either drift or selection. Rapid rates of evolution are also not unprecedented in pupfishes, and evolutionary divergence in thermal scope has already been documented in *C. nevadensis* populations (Hirshfield *et al*., 1980). Recent studies in pupfishes have also documented contemporary evolution of body shape (Collyer *et al*., 2007, 2011), and two adaptive radiations of pupfishes have been linked to exceptionally high rates of evolution in trophic morphology (Martin and Wainwright, 2011). In addition, population variation in TH physiology has been observed in other fishes and may be an important physiological mechanism underlying local population differentiation in energy metabolism or morphology (e.g. Kitano *et al*., 2010; Kitano and Lema, 2013; Levin and Bolotovskiy, 2015). Based on the differences in hepatic and muscle *dio* and *tr* expression observed here between the Amargosa River and Tecopa Bore *C. n. amargosae* populations, it is possible that variation in TH signaling, even if only at the level of some target tissues (e.g., Lema and Kitano, 2013), could contribute developmentally to the morphological variation between these populations.

### Conclusions

Conservation and management strategies for native desert fishes commonly aim to protected the species’ native habitat, which often consists of a single, small freshwater spring or marsh (Deacon and Minckley, 1991). Even for species where these habitats are currently protected, a changing climate may lead to altered temperatures and nutrient cycling in patterns that impact the energetics, growth and reproduction of native desert fishes (e.g. Hausner *et al*., 2014). For other desert fishes, habitat loss resulting from groundwater extraction or flow impediments, such as dams, has made it necessary to translocate fish to unoccupied habitats to establish additional populations or to breed fish in captivity for reintroduction into the wild (Johnson and Jensen, 1991; Minckley *et al*., 1991). Our finding here that endocrine and metabolic regulation by temperature differs in recently isolated (<50 years) populations of *C. n. amargosae* pupfish points to the importance of considering both contemporary population adaptation and thermal experience in management decisions about hatchery rearing conditions, reintroduction site selection and even fish translocation procedures in on-going efforts to conserve North America's native desert fishes.

## Supplementary Material

Supplementary Data

## References

[cow047C1] Arjona FJ, de Vrieze E, Visser TJ, Flik G, Klaren PHM (2011) Identification and functional characterization of zebrafish solute carrier Slc16a2 (Mct8) as a thyroid hormone membrane transporter. Endocrinology 152: 5065–5073. 21952246 10.1210/en.2011-1166

[cow047C2] Beitinger TL, Bennett WA, McCauley RW (2000) Temperature tolerances of North American freshwater fishes exposed to dynamic changes in temperature. Environ Biol Fish 58: 237–275.

[cow047C3] Blewett TA, MacLatchy DL, Wood CM (2013a) The effects of temperature and salinity on 17α-ethynylestradiol uptake and its relationship to oxygen consumption in the model euryhaline teleost (*Fundulus heteroclitus*). Aquat Toxicol 127: 61–71. 22575576 10.1016/j.aquatox.2012.04.009

[cow047C4] Blewett TA, Robertson LM, MacLatchy DL, Wood CM (2013b) Impact of environmental oxygen, exercise, salinity, and metabolic rate on the uptake and tissue-specific distribution of 17α-ethynylestradiol in the euryhaline teleost *Fundulus heteroclitus*. Aquat Toxciol 138–139: 43–51. 10.1016/j.aquatox.2013.04.006 23685400

[cow047C5] Boyle K-L, Dillaman RM, Kinsey ST (2003) Mitochondrial distribution and glycogen dynamics suggest diffusion constraints in muscle fibers of the blue crab, *Callinectes sapidus*. J Exp Zool A 297: 1–16. 10.1002/jez.a.10227 12911109

[cow047C6] Bozinovic F, Pörtner H-O (2015) Physiological ecology meets climate change. Ecol Evol 5: 1025–1030. 25798220 10.1002/ece3.1403 PMC4364817

[cow047C7] Bres O, Plohman JC, Eales JG (2006) A cDNA for a putative type III deiodinase in the trout (*Oncorhynchus mykiss*): influence of holding conditions and thyroid hormone treatment on its hepatic expression. Gen Comp Endocrinol 145: 92–100. 16214142 10.1016/j.ygcen.2005.07.008

[cow047C8] Brown DD (1997) The role of thyroid hormone in zebrafish and axolotl development. Proc Natl Acad Sci USA 94: 13011–13016. 9371791 10.1073/pnas.94.24.13011 PMC24254

[cow047C9] Brown JH, Feldmeth CR (1971) Evolution in constant and fluctuating environments: thermal tolerances of desert pupfish (*Cyprinodon*). Evolution 25: 390–398. 28563124 10.1111/j.1558-5646.1971.tb01893.x

[cow047C10] Bustin SA, Benes V, Garson JA, Hellemans J, Huggett J, Kubista M, Mueller R, Nolan T, Pfaffl MW, Shipley GL et al (2009) The MIQE guidelines: minimum information for publication of quantitative real-time PCR experiments. Clin Chem 55: 611–622. 19246619 10.1373/clinchem.2008.112797

[cow047C11] Caldwell ME, Slatoff LG, Lema SC (2015) Evidence for contemporary morphological diversification between populations of Amargosa pupfish. Integr Comp Biol 55: E228.

[cow047C12] Chatonnet F, Flamant F, Morte B (2015) A temporary compendium of thyroid hormone target genes in brain. Biochim Biophys Acta 1849: 122–129. 24882357 10.1016/j.bbagrm.2014.05.023

[cow047C13] Chown SL, Hoffmann AA, Kristensen TN, Angilletta MJ Jr, Stenseth NC, Pertoldi C (2010) Adapting to climate change: a perspective from evolutionary physiology. Clim Res 43: 3–15.

[cow047C14] Collin A, Joubert R, Swennen Q, Damon M, Métayer Coustard S, Skiba-Cassy S, Everaert N, Buyse J, Tesseraud S (2009) Involvement of thyroid hormones in the regulation of mitochondrial oxidations in mammals and birds In Kuehn FS, Lozada MP,eds, Thyroid Hormones: Functions, Related Diseases and Uses. Nova Science Publishers, Inc., New York, NY, pp 93–107.

[cow047C15] Collyer ML, Stockwell CA, Adams DC, Reiser MH (2007) Phenotypic plasticity and contemporary evolution in introduced populations: evidence from translocated populations of white sands pupfish (*Cyprinodon tularosa*). Ecol Res 22: 902–910.

[cow047C16] Collyer ML, Hellveil JS, Stockwell CA (2011) Contemporary evolutionary divergence for a protected species following assisted colonization. PLoS One 6: e22310. 21909348 10.1371/journal.pone.0022310 PMC3166134

[cow047C17] Darras VM, Houbrechts AM, Van Herck SLJ (2015) Intracellular thyroid hormone metabolism as a local regulator of nuclear thyroid hormone receptor-mediated impact on vertebrate development. Biochim Biophys Acta 1849: 130–141. 24844179 10.1016/j.bbagrm.2014.05.004

[cow047C18] Deacon JE, Minckley WL (1991) Western fishes and the real world: the enigma of ‘endangered species’ revisited In Minckley WL, Deacon JE,eds, Battle Against Extinction: Native Fish Management in the American West. University of Arizona Press, Tucson, AZ, pp 405–413.

[cow047C19] Dickhoff WW, Folmar LC, Mighell JL, Mahnken CVW (1982) Plasma thyroid hormones during smoltification of yearling and underyearling coho salmon and yearling Chinook salmon and steelhead trout. Aquaculture 28: 39–48.

[cow047C20] Eales JG, Chang JP, van der Kraak G, Omeijaniuk RJ, Uin L (1982) Effects of temperature on plasma thyroxine and iodine kinetics in rainbow trout, *Salmo gairdneri*. Gen Comp Endocrinol 47: 295–307. 7106551 10.1016/0016-6480(82)90237-4

[cow047C21] Fagan WF, Unmack PJ, Burgess C, Minckley WL (2002) Rarity, fragmentation, and extinction risk in desert fishes. Ecology 83:3250–3256.

[cow047C22] Feldmeth CR (1981) The evolution of thermal tolerance in desert pupfish (genus *Cyprinodon*) In Naiman J, Soltz DL,eds, Fishes of North American Deserts. John Wiley and Sons, New York, NY, pp 357–384.

[cow047C23] Ficke A, Myrick CA, Hansen LJ (2007) Potential impacts of global climate change on freshwater fisheries. Rev Fish Biol Fisheries 17: 581–613.

[cow047C24] Fields PA, Dong Y, Meng X, Somero GN (2015) Adaptations of protein structure and function to temperature: there is more than one way to ‘skin a cat’. J Exp Biol 218: 1801–1811. 26085658 10.1242/jeb.114298

[cow047C25] García-G C, Jeziorski MC, Valverde-R C, Orozco A (2004) Effects of iodothyronines on the hepatic outer-ring deiodinating pathway in killifish. Gen Comp Endocrinol 135: 201–209. 14697306 10.1016/j.ygcen.2003.09.010

[cow047C26] Ghazalpour A, Bennett B, Petyuk VA, Orozco L, Hagopian R, Mungrue IN, Farber CR, Sinsheimer J, Kang HM, Furlotte N et al (2011) Comparative analysis of proteome and transcriptome variation in mouse. PLoS Genet 7: e1001393. 21695224 10.1371/journal.pgen.1001393 PMC3111477

[cow047C27] Gil-Ibañez P, García-García F, Dopazo J, Bernal J, Morte B (2015) Global transcriptome analysis of primary cerebrocortical cells: identification of genes regulated by triiodothyronine in specific cell types. Cereb Cortex. doi:10.1093/cercor/bhv273. 10.1093/cercor/bhv273 26534908

[cow047C28] Hausner MB, Wilson KP, Gaines DB, Suárez F, Scoppettone GG, Tyler SW (2014) Life in a fishbowl: prospects for the endangered Devils Hole pupfish (*Cyprinodon diabolis*) in a changing climate. Water Resour Res 50: 7020–7034.

[cow047C29] Henry T, Ferguson A (1985) Kinetic studies on the lactate dehydrogenase (LDH-5) isozymes o brown trout, *Salmo trutta* L. Comp Biochem Physiol B 82: 95–98. 4053579 10.1016/0305-0491(85)90134-8

[cow047C30] Heuton M, Ayala L, Burg C, Dayton K, McKenna K, Morante A, Puentedura G, Urbina N, Hillyard S, Steinberg S et al (2015) Paradoxical anerobism in desert pupfish. J Exp Biol 218: 3739–3745. 26632453 10.1242/jeb.130633

[cow047C31] Hirshfield MF, Feldmeth CR, Soltz DL (1980) Genetic differences in physiological tolerances of Amargosa pupfish (*Cyprinodon nevadensis*) populations. Science 207: 999–1001. 17830462 10.1126/science.207.4434.999

[cow047C32] Hoffman AA, Sgrò CM (2011) Climate change and evolutionary adaptation. Nature 470: 479–485. 21350480 10.1038/nature09670

[cow047C33] Hofmann GE, Todgham AE (2010) Living in the now: physiological mechanisms to tolerate a rapidly changing environment. Ann Rev Physiol 72: 127–145. 20148670 10.1146/annurev-physiol-021909-135900

[cow047C34] Johnson JE, Jensen BL (1991) Hatcheries for endangered freshwater fishes In Minckley WL, Deacon JE,eds, Battle Against Extinction: Native Fish Management in the American West. University of Arizona Press, Tuscon, AZ, USA, pp 199–217.

[cow047C35] Johnson KM, Lema SC (2011) Tissue-specific thyroid hormone regulation of gene transcripts encoding iodothyroinine deiodinases and thyroid hormone receptors in striped parrotfish (*Scarus iseri*). Gen Comp Endocrinol 172: 505–517. 21549118 10.1016/j.ygcen.2011.04.022

[cow047C36] Johnson LK, Dillaman RM, Gay DM, Blum JE, Kinsey ST (2004) Metabolic influences of fiber size in aerobic and anaerobic locomotor muscles of the blue crab, *Callinectes sapidus*. J Exp Biol 207: 4045–4056. 15498950 10.1242/jeb.01224

[cow047C37] Johnston CE, Eales JG (1995) Effects of acclimation and assay temperature on outer-ring and inner-ring thyroxine and 3,5,3′-triiodo-l-thyronine deiodination by liver-microsomes of rainbow trout, *Oncorhynchus mykiss* J Exp Zool 272: 426–434.

[cow047C38] Jones RA, Cohn WB, Wilkes AA, MacKenzie DS (2016) Negative feedback regulation of thyrotropin subunits and pituitary deiodinases in red drum, *Sciaenops ocellatus*. Gen Comp Endocrinol 240: 19–26. 27597549 10.1016/j.ygcen.2016.09.003

[cow047C39] Kawakami Y, Shin D-H, Kitano T, Adachi S, Yamauchi K, Ohta H (2006) Transactivation activity of thyroid hormone receptors in fish (*Conger myriaster*) in response to thyroid hormone. Comp Biochem Physiol B 144: 503–509. 16820313 10.1016/j.cbpb.2006.05.003

[cow047C40] Kim D-K, Ryu D, Koh M, Lee M-W, Lim D, Kim M-J, Kim Y-H, Cho W-J, Lee C-H, Park SB et al (2012) Orphan nuclear receptor estrogen-related receptor γ (ERRγ) is key regulator of hepatic gluconeogenesis. J Biol Chem 287: 21628–21639. 22549789 10.1074/jbc.M111.315168 PMC3381127

[cow047C41] Kitano J, Lema SC (2013) Divergence in thyroid hormone concentrations between juveniles of marine and stream ecotypes of the threespine stickleback (*Gasterosteus aculeatus*). Evol Ecol Res 15: 143–153.

[cow047C42] Kitano J, Lema SC, Luckenbach JA, Mori S, Kawagishi Y, Kusakabe M, Swanson P, Peichel CL (2010) Adaptive divergence in the thyroid hormone signaling pathway in the stickleback radiation. Curr Biol 20: 2124–2130. 21093265 10.1016/j.cub.2010.10.050 PMC3053127

[cow047C43] Köhrl J (2000) The deiodinase family: selenoenzymes regulating thyorid hormone availability and action. Cell Mol Life Sci 57: 1853–1863. 11215512 10.1007/PL00000667 PMC11147027

[cow047C44] Layden A, Merchant C, MacCallum S (2015) Global climatology of surface water temperatures of large lakes by remote sensing. Intern J Climatol 35: 4464–4479.

[cow047C45] Leatherland JF, Cho CY, Slinger SJ (1977) Effects of diet, ambient temperature, and holding conditions on plasma thyroxine levels in rainbow trout (*Salmo gairdneri*). J Fish Res Board Can 34: 677–682.

[cow047C46] Leatherland JF, Cho CY, Hilton JW, Slinger SJ (1980) Further studies on the effect of diet on serum thyroid hormone concentrations and thyroid histology in rainbow trout, *Salmo gairdneri* (Pisces, Salmonidae). Environ Biol Fish 5: 175–179.

[cow047C47] Lee S, Ji K, Choi K (2014) Effects of water temperature on perchlorate toxicity to the thyroid and reproductive system of *Oryzias latipes*. Ecotoxicol Environ Safety 108: 311–317. 25108511 10.1016/j.ecoenv.2014.07.016

[cow047C48] Lema SC (2008) The phenotypic plasticity of Death Valley's pupfish. Am Sci 96: 28–36.

[cow047C49] Lema SC (2014) Hormones and phenotypic plasticity in an ecological context: linking physiological mechanisms to evolutionary processes. Integr Comp Biol 54: 850–863. 24752548 10.1093/icb/icu019

[cow047C50] Lema SC, Kitano J (2013) Hormones and phenotypic plasticity: implications for the evolution of integrated adaptive phenotypes. Curr Zool 59: 506–525.

[cow047C51] Lema SC, Nevitt GA (2006) Testing an ecophysiological mechanism for morphological plasticity in pupfish and its relevance to conservation efforts for endangered Devils Hole pupfish. J Exp Biol 209: 3499–3509. 16943490 10.1242/jeb.02417

[cow047C52] Lema SC, Dickey JT, Schultz IR, Swanson P (2009) Thyroid hormone regulation of mRNAs encoding thyrotropin β-subunit, glycoprotein α-subunit, and thyroid hormone receptors α and β in brain, pituitary gland, liver, and gonads of an adult teleost, *Pimephales promelas*. J Endocrinol 202: 43–54. 19380459 10.1677/JOE-08-0472

[cow047C53] Lema SC, Wagstaff LJ, Gardner NM (2010) Diurnal rhythms of behavior and brain mRNA expression for arginine vasotocin, isotocin, and their receptors in wild Amargosa pupfish (*Cyprinodon nevadensis amargosae*). Mar Fresh Behav Physiol 43: 257–281.

[cow047C54] Lema SC, Sander KE, Walti KA (2015) Arginine vasotocin, isotocin and nonapeptide receptor gene expression link to social status and aggression in sex-dependent patterns. J Neuroendocrinol 27: 142–157. 25425529 10.1111/jne.12239

[cow047C55] Lema SC, Wilson KP, Senger BL, Simons LH (2016) Sequencing and characterization of the complete mitochondrial genome of the endangered Devils Hole pupfish *Cyprinodon diabolis* (Cyprinodontiformes: Cyprinodontidae). Mitochondr DNA B 1: 705–707. 10.1080/23802359.2016.1225526 PMC7800719 33473600

[cow047C56] Levin BA, Bolotovskiy AA (2015) Discovery of latitudinal gradient of triiodothyronine concentrations in ecotherms as revealed from a cyprinid fish, the common roach *Rutilus rutilus*. Biochem Syst Ecol 62: 128–136.

[cow047C57] Little AG (2016) A review of the peripheral levels of regulation by thyroid hormone. J Comp Physiol B 186: 677–688. 27062031 10.1007/s00360-016-0984-2

[cow047C58] Little AG, Kunisue T, Kannan K, Seebacher F (2013) Thyroid hormone actions are temperature-specific and regulate thermal acclimation in zebrafish (*Danio rerio*). BMC Biol 11: 26. 23531055 10.1186/1741-7007-11-26 PMC3633057

[cow047C59] Lombardi A, Moreno M, de Lange P, Iossa S, Busiello RA, Goglia F (2015) Regulation of skeletal muscle mitochondrial activity by thyroid hormones: focus on the ‘old’ triiodothyronine and the ‘emerging’ 3,5-diiodothyronine. Front Physiol 6: 237. 26347660 10.3389/fphys.2015.00237 PMC4543916

[cow047C60] McCauley RW, Thomson DA (1988) Thermoregulatory activity in the Tecopa pupfish, *Cyprinodon nevadensis amargosae*, an inhabitant of a thermal spring. Environ Biol Fish 23: 135–139.

[cow047C61] McKim JM, Erikson RJ (1991) Environmental impacts on the physiological mechanisms controlling xenobiotic transfer across fish gills. Physiol Zool 64: 39–67.

[cow047C62] Maier T, Güell M, Serrano L (2009) Correlation of mRNA and protein in complex biological samples. FEBS Lett 583: 3966–3973. 19850042 10.1016/j.febslet.2009.10.036

[cow047C63] Martin CH, Crawford JE, Turner BJ, Simons LH (2016) Diabolical survival in Death Valley: recent pupfish colonization, gene flow and genetic assimilation in the smallest species range on Earth. Proc Biol Sci 283: 20152334. 26817777 10.1098/rspb.2015.2334 PMC4795021

[cow047C64] Martin CH, Wainwright PC (2011) Trophic novelty is linked to exceptional rates of morphological diversification in two adaptive radiations of *Cyprinodon* pupfish. Evolution 65: 2197–2212. 21790569 10.1111/j.1558-5646.2011.01294.x

[cow047C65] Miller RR (1948) The cyprinodont fishes of the Death Valley system of eastern California and southwestern Nevada. Misc Pub Mus Zool Univ Michigan 68: 1–155.

[cow047C66] Miller RR, Williams JD, Williams JE (1989) Extinctions of North American fishes during the past century. Fisheries 14: 22–38.

[cow047C67] Minckley WL, Meffe GK, Soltz DL (1991) Conservation and management of short-lived fishes: the cyprinodontoids In Minckley WL, Deacon JE,eds, Battle Against Extinction: Native Fish Management in the American West. University of of Arizona Press, Tucson, AZ, pp 247–282.

[cow047C68] Motani R, Wainwright PC (2015) How warm is too warm for the life cycle of actinopterygian fishes. Sci Rep 5: 11597. 26166622 10.1038/srep11597 PMC4648408

[cow047C69] Moyle PB, Katz JVE, Quiñones RM (2011) Rapid decline of California's native inland fishes: a status assessment. Biol Conserv 144: 2414–2423.

[cow047C70] Mullur R, Liu Y-Y, Brent GA (2014) Thyroid hormone regulation of metabolism. Physiol Rev 94: 355–382. 24692351 10.1152/physrev.00030.2013 PMC4044302

[cow047C71] Muzzio AM, Noyes PD, Stapleton HM, Lema SC (2014) Tissue distribution and thyroid hormone effects on mRNA abundance for membrane transporters Mct8, Mct10, and organic anion-transporting polypeptides (Oatps) in a teleost fish. Comp Biochem Physiol A Mol Integr Physiol 167: 77–89. 24113777 10.1016/j.cbpa.2013.09.019 PMC4160178

[cow047C72] Naiman RJ (1974) Bioenergetics of a pupfish population (*Cyprinodon*) and its algal food supply in a thermal stream. PhD dissertation, Arizona State University, Tempe, AZ.

[cow047C73] Nelson ER, Habibi HR (2006) Molecular characterization and sex-related seasonal expression of thyroid receptor subtypes in goldfish. Mol Cell Endocrinol 253: 83–95. 16777315 10.1016/j.mce.2006.05.003

[cow047C74] Nelson ER, Allan ERO, Pang FY, Habibi HR (2011) Auto-regulation of thyroid hormone receptors in the goldfish ovary and testis. Gen Comp Endocrinol 172: 50–55. 21187097 10.1016/j.ygcen.2010.12.017

[cow047C75] Noyes PD, Lema SC, Macaulay LJ, Douglas NK, Stapleton HM (2013) Low level exposure to the flame retardant BDE-209 reduces thyroid hormone levels and disrupts thyroid signaling in fathead minnows. Environ Sci Technol 47: 10012–10021. 23899252 10.1021/es402650x PMC3778448

[cow047C76] O'Reilly CM, Sharma S, Gray DK, Hampton SE, Read JS, Rowley RJ, Schneider P, Lenters JD, McIntyre PB, Kraemer BM et al (2015) Rapid and highly variable warming of lake surface waters around the globe. Geophys Res Lett 42: 773–781.

[cow047C77] Orozco A, Valverde RC (2005) Thyroid hormone deiodination in fish. Thyroid 15: 799–813. 16131323 10.1089/thy.2005.15.799

[cow047C78] Otto RG, Gerking SD (1973) Heat tolerance of a Death Valley pupfish (genus *Cyprinodon*). Physiol Zool 46: 43–49.

[cow047C79] Pillar TM, Seitz HJ (1997) Thyroid hormone and gene expression in the regulation of mitochondrial respiratory function. Eur J Endocrinol 136: 231–239. 9100544 10.1530/eje.0.1360231

[cow047C80] Place AR, Powers DA (1984) Kinetic characterization of the lactate dehydrogenase (LDH-B4) allozymes of *Fundulus heteroclitus*. J Biol Chem 259:1309–1318. 6693387

[cow047C81] Pörtner HO, Farrell AP (2008) Physiology and climate change. Science 322: 690–692. 18974339 10.1126/science.1163156

[cow047C82] Psarra A-MG, Sekeris CE (2013) Steroid and thyroid hormone receptors in mitochondria. IUBMB Life 60: 210–223. 10.1002/iub.37 18344181

[cow047C83] Psarra A-MG, Solakidi S, Sekeris CE (2006) The mitochondrion as a primary site of action of steroid and thyroid hormones: presence and action of steroid and thyroid hormone receptors in mitochondria of animal cells. Mol Cell Endocrinol 246: 21–33. 16388892 10.1016/j.mce.2005.11.025

[cow047C84] Quattro JM, Woods HA, Powers DA (1993) Sequence analysis of teleost retina-specific lactate dehydrogenase C: evolutionary implications for the vertebrate lactate dehydrogenase gene family. Proc Natl Acad Sci USA 90: 242–246. 8419929 10.1073/pnas.90.1.242 PMC45636

[cow047C85] Rodríquez-Peña A, Escrivá H, Handler AC, Vallejo CG (2002) Thyroid hormone increases transcription of GA-binding protein/nuclear respiratory factor-2 α-subunit in rat liver. FEBS Lett 514: 309–314. 11943172 10.1016/s0014-5793(02)02389-x

[cow047C86] Roessig JM, Woodley CM, Cech JJ Jr, Hansen LJ (2004) Effects of global climate change on marine and estuarine fishes and fisheries. Rev Fish Biol Fish 14: 251–275.

[cow047C87] St Germain DL, Glaton VA, Hernandez A (2009) Defining the roles of iodothyronine deiodinases: current concepts and challenges. Endocrinology 150: 1097–1107. 19179439 10.1210/en.2008-1588 PMC2654746

[cow047C88] Schneider P, Hook SJ, Radocinski RG, Corlett GK, Hulley GC, Schladow SG, Steissberg TE (2009) Satellite observations indicate rapid warming trend for lakes in California and Nevada. Geophys Res Lett 36: L22402.

[cow047C89] Schweizer U, Weitzel JM, Schomburg L (2008) Think globally: act locally. New insights into the local regulation of thyroid hormone availability challenge long accepted dogmas. Mol Cell Endocrinol 289: 1–9. 18508193 10.1016/j.mce.2008.04.007

[cow047C90] Seager R, Vecchi GA (2010) Greenhouse warming and the 21st century hydroclimate of southwestern North America. Proc Natl Acad Sci USA 107: 21277–21282. 21149692 10.1073/pnas.0910856107 PMC3003097

[cow047C91] Seager R, Ting M, Held I, Kushnir Y, Lu J, Vecchi G, Huang H-P, Harnik N, Leetmaa A, Lau N-C et al (2007) Model predictions of an imminent transition to a more arid climate in southwestern North America. Science 316: 1181–1184. 17412920 10.1126/science.1139601

[cow047C92] Silva JE (1995) Thyroid hormone control of thermogenesis and energy balance. Thyroid 5: 481–492. 8808101 10.1089/thy.1995.5.481

[cow047C93] Soltz DL, Naiman RJ (1978) The natural history of native fishes in the Death Valley system. Nat Hist Mus Los Angeles County, Sci Ser 30: 1–76.

[cow047C94] Suarez RK, Moyes CD (2012) Metabolism in the age of ‘omes’. J Exp Biol 215: 2351–2357. 22723473 10.1242/jeb.059725

[cow047C95] Tyagi S, Gupta P, Saini AS, Kaushal C, Sharma S (2011) The peroxisome proliferator-activated receptor: a family of nuclear receptors role in various diseases. J Adv Phar Technol Res 2: 236–240. 10.4103/2231-4040.90879 PMC3255347 22247890

[cow047C96] Vaitkus JA, Farrar JS, Celi FS (2015) Thyroid hormone mediated modulation of energy expenditure. Int J Mol Sci 16: 16158–16175. 26193258 10.3390/ijms160716158 PMC4519944

[cow047C97] Villena JA, Hock MB, Chang WY, Barcas JE, Giguère V, Kralli A (2007) Orphan nuclear receptor estrogen-related receptor α is essential for adaptive thermogenesis. Proc Natl Acad Sci USA 104: 1418–1423. 17229846 10.1073/pnas.0607696104 PMC1783094

[cow047C98] Visser WE, Friesema ECH, Visser TJ (2011) Thyroid hormone transporters: the knowns and the unknowns. Mol Endocrinol 25: 1–14. 20660303 10.1210/me.2010-0095 PMC5417302

[cow047C99] Walsh PJ, Henry RP (1990) Activities of metabolic enzymes in the deep-water crabs *Chaceon fenneri* and *C. quinquedens* and the shallow-water crab *Callinectes sapidus*. Mar Biol 106: 343–346.

[cow047C100] Weitzel JM, Iwen KA (2011) Coordination of mitochondrial biogenesis by thyroid hormone. Mol Cell Endocrinol 342: 1–7. 21664416 10.1016/j.mce.2011.05.009

[cow047C101] Weitzel JM, Iwen KAH, Seitz HJ (2003) Regulation of mitochondrial biogenesis by thyroid hormone. Exp Physiol 88: 121–128. 12552316 10.1113/eph8802506

[cow047C102] Wood CM, McDonald DG (1997) Global Warming: Implications for Freshwater and Marine Fish. Cambridge University Press, Cambridge, UK, pp 1–425.

[cow047C103] Wrutniak-Cabello C, Casas F, Cabello G (2001) Thyroid hormone action in mitochondria. J Mol Endocrinol 26: 67–77. 11174855 10.1677/jme.0.0260067

[cow047C104] Yamanoue Y, Setiamarga DHE, Matsuura K (2010) Pelvic fins in teleosts: structure, function and evolution. J Fish Biol 77: 1173–1208. 21039499 10.1111/j.1095-8649.2010.02674.x

[cow047C105] Yen PM (2001) Physiological and molecular basis of thyroid hormone action. Physiol Rev 81: 1097–1142. 11427693 10.1152/physrev.2001.81.3.1097

[cow047C106] Yen PM, Ando S, Feng X, Liu Y, Maruvanda P, Xia X (2006) Thyroid hormone action at the cellular, genomic and target gene levels. Mol Cell Endocrinol 246: 121–127. 16442701 10.1016/j.mce.2005.11.030

