# Supplementary material for: Endocrine and metabolic impacts of warming aquatic habitats: differential responses between recently isolated populations of a eurythermal desert pupfish
Source: Conserv Physiol. 2016 Nov 3;4(1):cow047. doi: 10.1093/conphys/cow047 (PMC5100229; doi:10.1093/conphys/cow047)
Supplement: Supplementary Data [file cow047_conphys-2016-032.pdf]

**Table S1.** Gene-specific primers for SYBR green quantitative PCR in Amargosa pupfish.

| Transcript | Primer | Nucleotide Sequence (5′ to 3′) | Amplicon length (bp) | % efficiency (avg.) | GenBank accession no. |
|---|---|---|---|---|---|
| *dio1* | for | GAG AAC CAG CAT CTA CCA ATA CA | 114 bp | 102.26% | KT879790 |
| | rev | CTG ACG AGC TGC TTG AAC T | | | |
| *dio2* | for | CTT CAG TGA GGT GGC TGA TTT | 123 bp | 102.55% | KT879791 |
| | rev | TCC TCA AGG TTC TGA TGT TTC C | | | |
| *dio3* | for | CCA GAA GCT GGA CTT TCT CAA | 124 bp | 99.53% | KT879792 |
| | rev | CAG CGG TCT CTT CCC TTT C | | | |
| *trαA* | for | CTG CCT TGT GAA GAC CAG ATT A | 94 bp | 100.53% | KT879793 |
| | rev | TTT CGC TTT CAG GGT CGT AG | | | |
| *trαB* | for | CAT GCA GCA GCC TTT CAA TAA G | 127 bp | 101.16% | KT879794 |
| | rev | CTT CAG CGA GTT CAC CAA GAT | | | |
| *trβ* | for | ACC ATC CAG AAG AAC CTG AAC | 108 bp | 101.50% | KT879795 |
| | rev | GCA CTT CTT GAA GCG ACA TTC | | | |
| *mct8* | for | GCC TCA CAA GCC ATA GGT TA | 96 bp | 101.10% | JSUU01001843* |
| | rev | GTT ACC GAA GTA GCC ATG AAG A | | | |
| *oatp1c1* | for | CCT TAT AGC TGG CGT CAT AAC T | 95 bp | 99.35% | JSUU01029502* |
| | rev | CCT TCT TTG GGA CCC TTG AA | | | |
| *atpase6/8* | for | GGT CAC AAG TGA GCC CTT ATT | 102 bp | 102.85% | KU883631 |
| | rev | TAG CTG GGT GGT AGG TGT AA | | | |
| *cox2* | for | GAC TAC GAA GAC CTC GGA TTT G | 94 bp | 98.61% | KU883631 |
| | rev | CCA TTC GGT GGT CTG TTT CT | | | |
| *cs* | for | CAA AGA GTG GGC GAA GAG A | 106 bp | 105.46% | JSUU01011504* |
| | rev | TGT GAT GGC AGC ACT GAA | | | |
| *ldhA* | for | AAG CTC AGT GGC TTC CC | 93 bp | 102.03% | JSUU01000449* |
| | rev | GTG GAA CTT CTC TCC CAT GAT A | | | |
| *ldhB* | for | GGA GGA CCG CCT CAA AG | 83 bp | 105.85% | JSUU01016800* |
| | rev | CTT TAT CCG CAA CCA CCT TG | | | |
| *ldhC* | for | CGA CTT AAC TGA GAG CCT GAT G | 104 bp | 103.62% | JSUU01009679* |
| | rev | GCA GAC TCA GGT ATA CTT CTT CAC | | | |
| *atpaseA* | for | GAT GGA ACC GAC GAG AAG AAA | 102 bp | 104.64% | JSUU01012053* |
| | rev | ATC TGC ATC AGT TAG CCT CTT C | | | |
| *atpaseB* | for | GCT GTC TGA GGA AGA CAA ACT | 102 bp | 101.39% | JSUU01013195* |
| | rev | CCC AAG TGA CCT GTG AAG AC | | | |
| *cox5b* | for | GGT TCC CTC CAT CAC TAA CAA G | 92 bp | 99.54% | JSUU01005140* |
| | rev | CAT GAA GCC AGA ACC AGA C | | | |
| *nrf-1* | for | CAG CAT GTA CCA GAC TGT AGT G | 106 bp | 101.76% | JSUU01010574* |
| | rev | GTC AAG GGT GAC CGT GTT T | | | |
| *pparα* | for | CCA GAT GAC GCT TTC CTC TT | 99 bp | 103.52% | JSUU01009178* |
| | rev | CTG TTG TCC TGA TGT CCT GAA | | | |
| *pparδ* | for | GCC CTG GTT TAA TGA ACG TAA AG | 110 bp | 103.01% | JSUU01009630* |
| | rev | GGG AAA GAG GTA GAG AGA GTC A | | | |
| *esrra* | for | CGT TCC CAT GCA CAA ACT ATT C | 75 bp | 97.55% | JSUU01002554* |
| | rev | GTC TTT CGC TCC ATC CTC TAA G | | | |
| *esrrga* | for | GAG GCT TCA AGA TGT CCT TCA | 97 bp | 104.35% | JSUU01002667* |
| | rev | GAG AGT CAT GAT CAG CTT TCC A | | | |
| *ef-1α* | for | CCT GGG TAT TGG ACA AAC TGA | 90 bp | 100.00% | EU906930 |
| | rev | CGT AGT ACT TGC TGG TCT CAA A | | | |
| *rpl8* | for | GAC CAA GAA GTC CAG AGT CAA G | 116 bp | 99.42% | KT719257 |
| | rev | TCA GGA TGG GCT TGT CAA TAC | | | |

Note: * indicates Whole Genome Shotgun Sequence from closely-related *C. n. pectoralis* pupfish.

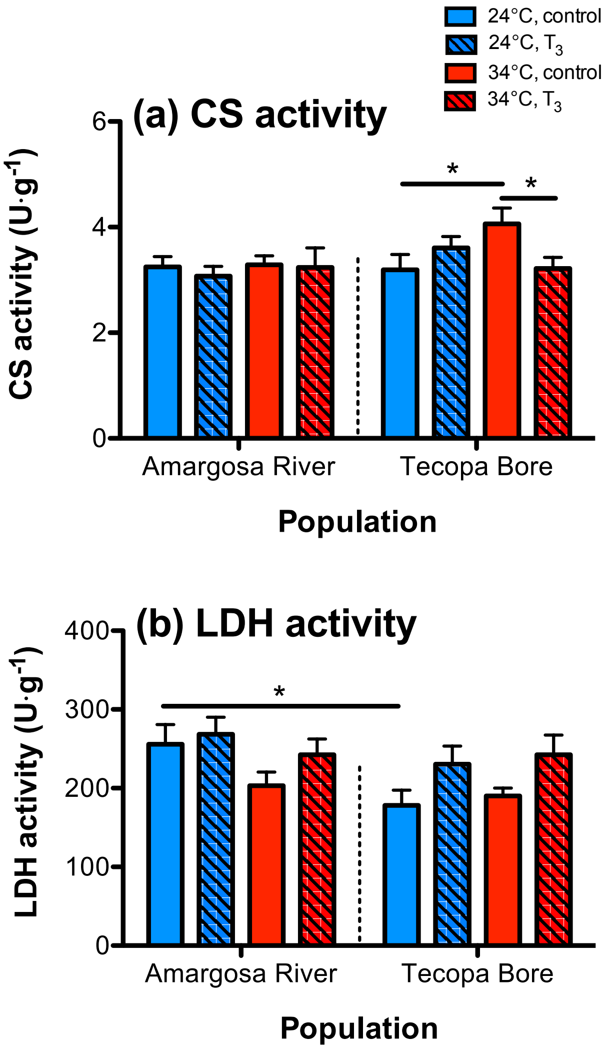


**Figure S1.** Citrate synthase (CS) and lactate dehydrogenase (LDH) enzyme activity in liver. (**a**) CS activity was elevated in Tecopa Bore pupfish to 34°C compared to 24°C, although no temperature effect was observed in the Amargosa River population. $T_3$ reduced hepatic CS activity in Tecopa Bore pupfish at 34°C, but not at 24°C. $T_3$ had no effect on CS activity in Amargosa River fish. (**b**) Hepatic LDH activity varied between the populations, with elevated CS activity in Amargosa River pupfish at 24°C compared to Tecopa Bore fish at 24°C. $T_3$ elevated LDH activity in both populations (ANOVA, $F_{1,78} = 5.846$, $p = 0.0179$), although this hormone effect was not apparent in *post hoc* pairwise comparisons. Data from males and females are combined. Lines denote pairwise differences (planned *t* tests: * $p < 0.025$).

**Figure S2**

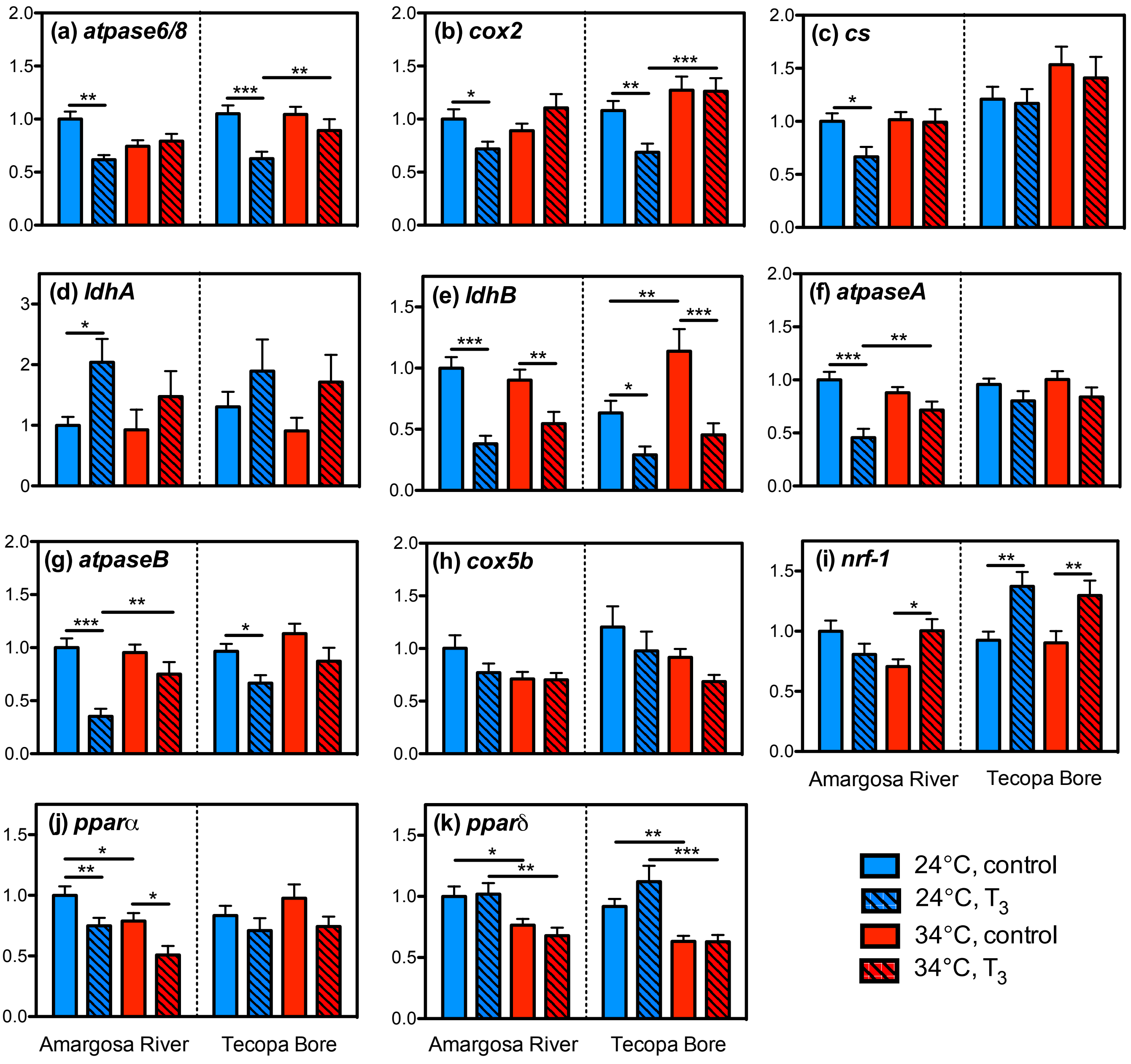


**Figure S2.** Population, temperature, and $T_3$ effects on liver metabolic gene transcript expression. Relative mRNA levels for (**a**) mitochondrial *atpase6/8*, (**b**) mitochondrial *cox2*, (**c**) *cs*, (**d**) *ldhA*, (**e**) *ldhB*, (**f**) *atpaseA*, (**g**) *atpaseB*, (**h**) *cox5b*, (**i**) *nrf-1*, (**j**) *ppar* , and (**k**) *ppar* in the liver of male and female (combined) pupfish from the Amargosa River and Tecopa Bore populations. Note that scales of y-axis relative mRNA levels differ among transcripts. Lines indicate pairwise differences between groups (planned *t* tests: * $p < 0.025$, ** $p < 0.01$, *** $p < 0.001$). Data from males and females are shown combined, and sample sizes are $n$ =14-17 fish per treatment group.

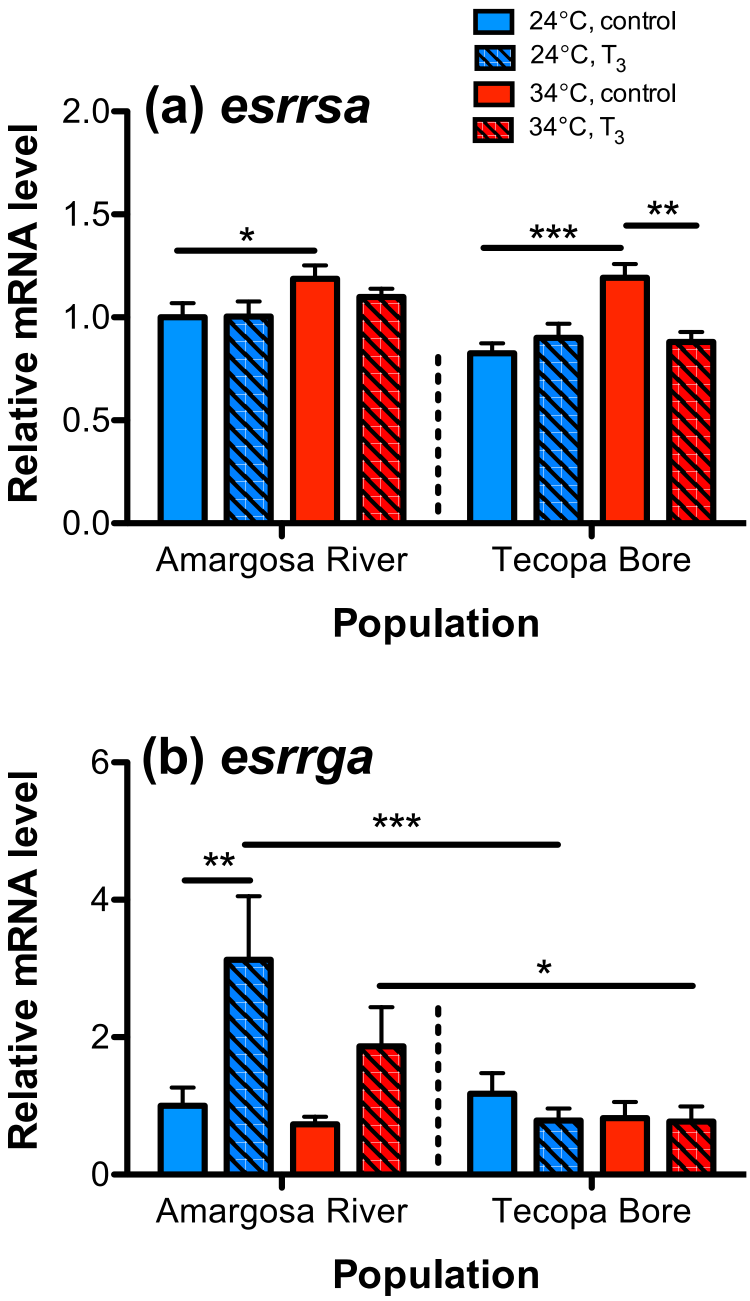


**Figure S3.** Relative abundance of mRNAs encoding estrogen-related receptors EER (*eersa*) and EER (*esrrga*) in liver. (**a**) Transcripts for *eersa* were elevated in pupfish acclimated to to 34°C compared to those at 24°C. Tecopa Bore pupfish exposed to exogenous $T_3$ exhibited reduced hepatic *eersa* mRNA levels compared to control fish. (**b**) Liver *esrrga* mRNA levels were elevated 2- to 3-fold by $T_3$ in the Amargosa River population, but not in the Tecopa Bore population. Data from males and females are shown combined. Lines indicate significant differences (planned *t* tests: $p < 0.025$, ** $p < 0.01$, *** $p < 0.001$), and $n$ =14-17 fish per treatment group.
